# Targeting mTOR as a Cancer Therapy: Recent Advances in Natural Bioactive Compounds and Immunotherapy

**DOI:** 10.3390/cancers14225520

**Published:** 2022-11-10

**Authors:** Abdelhakim Bouyahya, Aicha El Allam, Sara Aboulaghras, Saad Bakrim, Naoual El Menyiy, Mohammed Merae Alshahrani, Ahmed Abdullah Al Awadh, Taoufiq Benali, Learn-Han Lee, Nasreddine El Omari, Khang Wen Goh, Long Chiau Ming, Mohammad S. Mubarak

**Affiliations:** 1 Laboratory of Human Pathologies Biology, Department of Biology, Faculty of Sciences, Faculty of Medicine and Pharmacy, Mohammed V University in Rabat, Rabat 10106, Morocco; 2 Department of Immunology, Yale University School of Medicine, 333 Cedars Street, TAC S610, New Haven, CT 06519, USA; 3 Physiology and Physiopathology Team, Faculty of Sciences, Genomic of Human Pathologies Research, Mohammed V University in Rabat, Rabat 10106, Morocco; 4 Geo-Bio-Environment Engineering and Innovation Laboratory, Molecular Engineering, Biotechnologies and Innovation Team, Polydisciplinary Faculty of Taroudant, Ibn Zohr University, Agadir 80000, Morocco; 5 Laboratory of Pharmacology, National Agency of Medicinal and Aromatic Plants, Taounate 34025, Morocco; 6 Department of Clinical Laboratory Sciences, Faculty of Applied Medical Sciences, Najran University, 1988, Najran 61441, Saudi Arabia; 7 Environment and Health Team, Polydisciplinary Faculty of Safi, Cadi Ayyad University, Sidi Bouzid B.P. 4162, Morocco; 8 Novel Bacteria and Drug Discovery Research Group (NBDD), Microbiome and Bioresource Research Strength (MBRS), Jeffrey Cheah School of Medicine and Health Sciences, Monash University Malaysia, Bandar Sunway 47500, Malaysia; 9 Laboratory of Histology, Embryology, and Cytogenetic, Faculty of Medicine and Pharmacy, Mohammed V University, Rabat 10100, Morocco; 10 Faculty of Data Science and Information Technology, INTI International University, Nilai 71800, Malaysia; 11 Pengiran Anak Puteri Rashidah Sa’adatul Bolkiah Institute of Health Sciences, Universiti Brunei Darussalam, Gadong BE1410, Brunei; 12 Department of Chemistry, The University of Jordan, Amman 11942, Jordan

**Keywords:** mTOR, immunotherapy, cancer, natural drugs, mTOR inhibitors

## Abstract

The mammalian target of rapamycin (mTOR) is a highly conserved serine/threonine-protein kinase, which regulates many biological processes related to metabolism, cancer, immune function, and aging. It is an essential protein kinase that belongs to the phosphoinositide-3-kinase (PI3K) family and has two known signaling complexes, mTOR complex 1 (mTORC1) and mTOR complex 2 (mTORC2). Even though mTOR signaling plays a critical role in promoting mitochondria-related protein synthesis, suppressing the catabolic process of autophagy, contributing to lipid metabolism, engaging in ribosome formation, and acting as a critical regulator of mRNA translation, it remains one of the significant signaling systems involved in the tumor process, particularly in apoptosis, cell cycle, and cancer cell proliferation. Therefore, the mTOR signaling system could be suggested as a cancer biomarker, and its targeting is important in anti-tumor therapy research. Indeed, its dysregulation is involved in different types of cancers such as colon, neck, cervical, head, lung, breast, reproductive, and bone cancers, as well as nasopharyngeal carcinoma. Moreover, recent investigations showed that targeting mTOR could be considered as cancer therapy. Accordingly, this review presents an overview of recent developments associated with the mTOR signaling pathway and its molecular involvement in various human cancer types. It also summarizes the research progress of different mTOR inhibitors, including natural and synthetised compounds and their main mechanisms, as well as the rational combinations with immunotherapies.

## 1. Introduction

mTOR represents the target of a molecule, known as sirolimus or rapamycin, produced by *Streptomyces hygroscopicus* [1]. This macrolide represents a highly conserved serine/threonine-protein kinase, which integrates multiple signals from extracellular and intracellular signaling pathways responsible for the regulation of many fundamental cells and biological processes related to metabolism, autophagy, mRNA translation, cell growth, survival, ribosome biogenesis, immune function, obesity, diabetes, and aging [2]. mTOR is an atypical protein kinase belonging to the phosphoinositide-3-kinase (PI3K) family and is commonly organized into two known signaling complexes, mTOR complex 1 (mTORC1) and mTOR complex 2 (mTORC2) [3]. The three main mTORC1 elements are mTOR, raptor (mTOR-associated regulatory protein), and mLST8 (mammalian lethal protein 8 with Sec13, also known as GβL). Additionally, mTORC1 consists of the two inhibitory subunits: proline-rich Akt substrate of 40 kDa (PRAS40) and DEP domain-containing mTOR-interacting protein (DEPTOR). In contrast, mTORC2 consists of mLST8, mTOR, rictor (an mTOR-independent raptor companion), and mSIN1 (mammalian stress-activated protein kinase interaction protein 1), protor-1/2 (observed with rictor protein composition-1/2), and DEPTOR [2]. While these two complexes share the same catalytic-TOR subunit, they phosphorylate distinct downstream targets and exhibit different cellular functions.

With the cooperation of the critical elements of mTORC1 and mTORC2, the mTOR signaling system is able to catalyze the phosphorylation of many targets, namely type-I insulin-like growth factor receptor (IGF-IR), protein kinase C (PKC), protein kinase B (Akt), eukaryotic translation initiation factor 4E binding protein 1 (4E-BP1), and ribosomal protein S6 kinase β-1 (S6K1). Thus, mTOR promotes mitochondria-related protein synthesis, suppresses the catabolic process of autophagy, contributes to lipid metabolism, engages in ribosome formation, and acts as a key regulator of mRNA translation [4,5,6]. Although mTOR signaling plays several evolutionarily conserved roles, it remains one of the signaling systems most involved in the tumor process, such as cell cycle, cell proliferation, and apoptosis. Indeed, mTORC1 promotes cancer genesis and metastasis by promoting autophagy and reversing glycolysis.

The mTOR signaling pathway may be proposed as a cancer biomarker, and its targeting constitutes a major therapeutic challenge. Indeed, the mTOR signaling dysregulation is involved in multiple human cancers, including lung, breast, cervical, colon, reproductive, and bone cancers, as well as nasopharyngeal carcinoma. Although previous work has highlighted the role of mTOR in the process of cancerization, the specific involvement of mTOR signaling in the different types of cancer, as well as the pathways of mTOR inhibition, remain poorly discussed. In this context, to determine new pharmacological approaches, researchers have already initiated the targeting of this pathway by using mTOR inhibitors alone or combined with therapies. In combination with natural compounds, mTOR inhibitors were used as a promising source of effective anticancer drugs such as apigetrin, dihydromyricetin, piperlongumine, thymoquinone, glycyrrhizic acid, cryptotanshinone, cannabisin B, licochalcone A, and curcumin [7]. These agents affect the modulation of autophagy via PI3K/Akt/mTOR pathway inhibition in different cancer cells [8]. Synthetic compounds also act as cancer-preventive therapeutics targeting the mTOR signaling pathway.

On the other hand, immunotherapy has attracted much attention to the development of targeted therapies inhibiting the PI3K-AKT-mTOR signaling network, which is dysfunctional in several forms of cancer. Recent studies have shown that mTOR plays a crucial role in immune system modulation. Interestingly, mTOR regulates T cells, tumor-associated macrophages (TAMs), and antigen-presenting cells and promotes their development and activation [9]. In addition, studies have demonstrated the modulation effects of mTORC1/2 on various human cells such as CD4, CD8, Treg, TAMs, cancer-associated fibroblasts (CAF), endothelial cells, and myeloid-derived suppressor cells (MDSCs) [10,11]. Based on these findings, there is increasing evidence that targeting the PI3K-AKT-mTOR signaling pathway may affect host immunity and cancer cells. Clinical advances in cancer immunotherapies that target immune checkpoint receptors (CTLA-4 and PD-1) have shown the relevance of immunoevasion as a specificity of malignancy and cancer [12,13]. In light of the previous discussion, this article reviews the current knowledge on the mTOR signaling pathway by highlighting its structure, various functions, and the upstream and downstream signaling related to cancer. In addition, this review summarizes the literature relating to the potential combination of mTOR inhibitors with several therapies for the management of various forms of cancer. Moreover, we provide a comprehensive update on the mechanisms underlying the effects of combining mTOR inhibitors with synthetic and natural compounds as cancer preventive and therapeutic agents.

## 2. mTOR Signaling Pathways

### 2.1. Structure of mTOR

mTOR is the target of a molecule called rapamycin or sirolimus. It is a macrolide produced by *Streptomyces hygroscopicus*. It first attracted attention due to its extensive antiproliferative properties. Mammalian biochemical methods led to mTOR purification and its discovery as a physical target of rapamycin [1,14,15]. Indeed, serine/threonine kinase mTOR is part of a family of phosphoinositide 3-kinase (PI3K)-related kinases (PIKK). This conserved protein integrates various upstream signals to regulate growth-related processes, including mRNA translation, ribosome biogenesis, autophagy, and metabolism [2]. Human mTOR is encoded by a highly conserved DNA sequence containing 166,963 base pairs and is located at 1p36.22 (NCBI gene ID: 2475). The mTOR gene expresses mature mTOR after transcription, splicing, translation, and modification. Mature mTOR, comprising 2549 amino acid residues, has a molecular weight of 289 kDa, and is at least 95% similar to mice and rats in terms of amino acid sequence [16].

In addition, mTOR forms the catalytic core of two known signaling complexes, mTORC1 and mTORC2 [3,17]. mTORC1 promotes anabolic cell metabolism in response to growth factors, nutrients, and energy and acts as the primary controller of cell growth. Although little is understood about mTORC1, mTORC2 responds to growth factors and controls cell metabolism, survival, and actin cytoskeleton organization. mTOR plays a vital role in cellular processes related to tumorigenesis, metabolism, immune function, and aging [6]. mTORC1 is composed of mTOR, raptor (a protein associated with mTOR regulation), PRAS40 (a 40 kDa proline-rich AKT substrate), mLST8; also known as GβL; (a mammalian lethal with sec-13), and mSIN1 (mammalian stress-activated protein kinase interaction protein 1) [2]. Its main functions are the control of cell proliferation and survival by phosphorylation and activation of Akt/PKB kinase [2]. mTORC1 and mTORC2 are characterized by the accessory proteins raptor and rictor, respectively. Rapamycin is a mTORC1 inhibitor that binds to the FK506-binding 12 kDa protein (FKBP12) and inhibits the raptor-bound rapamycin proteins but not mTOR [4].

**Figure 1:**
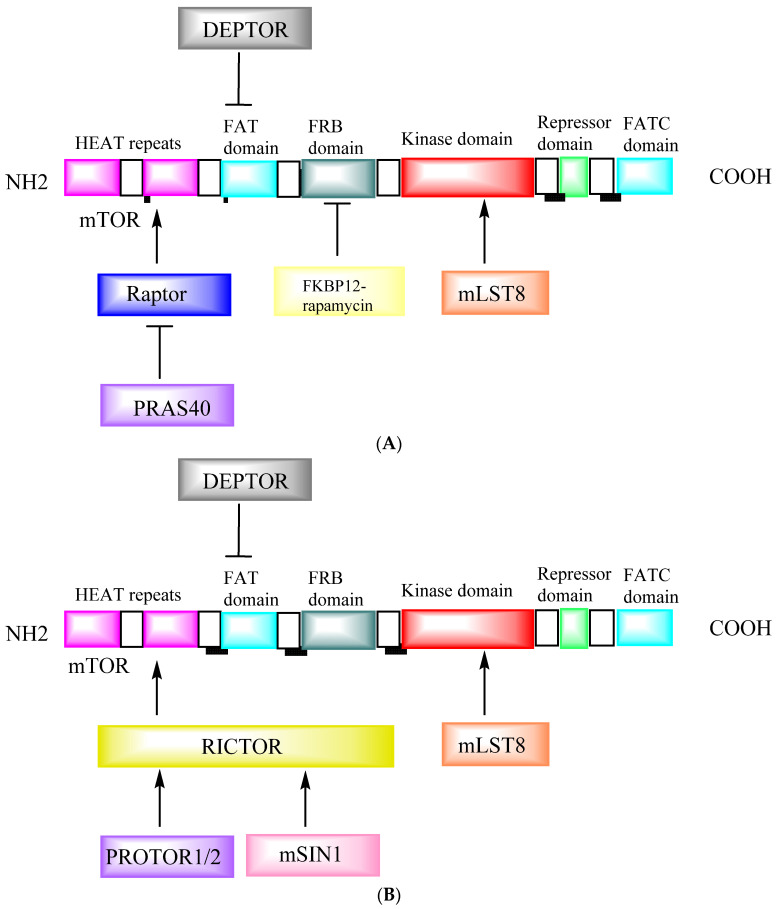
The structural domains of mTOR are shown hierarchically in this schematic. The HEAT domain, which is comprised of Huntingtin, elongation factor 3, subunit A of PP2A, and Tor1p; the FAT domain, which is comprised of FRAP-ATM-TRAPP; the FRB domain, which is comprised of FKBP12-rapamycin binding; and the FATC domain are the evolutionarily conserved structural domains of mTOR (FAT C-terminus). There are a total of 2549 amino acids found in the entire polypeptide sequence of mTOR. (**A**) Constituents of mTORC1: Important to the complex is the mTOR-interacting protein with the DEP domain (mLST8). The protein DEPTOR (mTOR-interacting protein) functions as an endogenous inhibitor of mTORC1. The mTOR-regulatory associated protein (Raptor), which has its own HEAT repeats and binds to mTOR, is the essential component of mTORC1. Moreover, in response to insulin, raptor recruits PRAS40, a proline-rich 40 kDa AKT substrate that suppresses mTORC1 activity. (**B**) mLST8, DEPTOR, and RICTOR, the defining component of mTORC2, are subunits of mTORC2. The rictor protein interacts with rictor-associated protein 1 and 2, which act as scaffolding proteins (RICTOR 1). Addition of PROTOR1/2, a pleckstrin homology domain-containing protein, and mSIN1, a MAPK interaction protein, to the complex.

The unique component of mTORC1 is raptor (regulatory-associated protein of mTOR). Raptor is a multidomain protein with an N-terminal RNC (raptor N-terminal conserved) domain, a regulatory domain, and numerous central HEAT repeats (called after hunting). HEAT repeats [huntingtin, elongation factor 3, the regulatory A subunit of PP2A (protein phosphatase 2A) and TOR1 (target of rapamycin 1)] and a WD40-propeller at the C-terminus [18,19]. Raptor plays a role in mTORC1 assembly and substrate binding [20,21]. The rictor subunits of mTORC2 are its distinguishing characteristics (rapamycin-insensitive companion of mTOR). The translation regulators 4EBP1 (eukaryotic translation initiation factor 4E binding protein 1) and S6K1 (ribosomal S6 kinase 1) are downstream effectors of the mTORC1 pathway [22]. The discovery of two structurally and functionally different multiprotein complex structures, including mTOR and TSC1/2, rheb, and AMP-activated protein kinase (AMPK) as upstream regulators of mTOR reveals how mTOR can perceive and respond to a variety of signals [2]. Figure 1 shows a schematic representation of the structural domains of mTOR.

### 2.2. Role of mTOR

#### 2.2.1. Role of mTORC1

The mTORC1 protein found in trophoblasts acts as a positive regulator of the placental amino acid transporter regulator, which causes it to influence the transfer of amino acids across the placenta. Therefore, placental mTORC1 signaling is an important connection between the availability of nutrients for the mother and the development of the fetus, which ultimately affects the health of the fetus over the long run. In addition, placental mTORC1 is regulated in pregnancy difficulties linked to stunted fetal growth as well as animal models in which the maternal food supply has been artificially changed [23]. Several signals controlling mTORC1 do this by altering the GTP-binding state of Rheb by activating or inhibiting the TSC-TBC complex, a GTPase-activating protein complex composed of TSC1, TSC2, and TBC1D7 [14]. In this respect, insulin, IGF1, and other growth hormones, for example, block the complex’s ability to activate Rheb and mTORC1 via Akt-mediated phosphorylation of TSC2 [24,25]. This complex is activated in response to a drop in cellular ATP, such as that seen during glucose depletion, and it partially inhibits Rheb and mTORC1 via AMPK activity.

When activated, mTORC1 phosphorylates S6K1 and S6K2, 4E-BP1, 4E-BP2, and many other downstream targets [3]. Therefore, mTORC1 signaling promotes anabolic cell proliferation while inhibiting the catabolic process of autophagy, an essential function that has been maintained throughout evolution. Multiple upstream signals that regulate the growth factor mTORC1 have been investigated in depth. By binding to specific receptor tyrosine kinases, growth factors activate the PI3K-Akt and Ras-mitogen-activated protein kinase (MAPK) pathways [23]. These pathways predominantly activate mTORC1 by phosphorylating and inhibiting tuberous sclerosis complex 2 (TSC2; also known as tuberin [24,25,26,27,28], which in combination with TSC1 (also known as hamartin) and TBC, (Tre2-Bub2-Cdc16). In addition, the TSC complex promotes Rheb’s intrinsic GTPase activity, which speeds up the pace at which Rheb switches between its active GTP-bound state and its inactive GDP-bound state. A rise in the ratio of Rheb-GTP to Rheb-GDP triggered by growth stimuli inhibits TSC2 GAP activity and activates mTORC1 [29].

#### 2.2.2. Autophagy and mTOR

Work published by Avivar-Valderas et al. [30] demonstrated that PERK kinase induces autophagy in ECM detached cells by activating AMPK, which inhibits mTORC1-p70 S6K signaling downstream. Moreover, forced activation of PERK or AMPK drives luminal filling and autophagy during acinar morphogenesis by maintaining a population of anoikis-resistant autophagic luminal cells. The activation of autophagy is negatively controlled by mTORC1, which is regulated by the insulin/insulin-like growth factor (IGF-1)-PI3K (phosphoinositide 3-kinase class I)-Akt signaling pathway. PTEN and TSC2 are negative mTORC1 signaling regulators, whereas PDK1 and Rheb, positive mTORC1 signaling regulators, are involved in the insulin/IGF-1 pathway. In this context, TSC2 and TSC1 function as a GTPase activator protein (GAP) for the small GTPase Rheb, with GAP activity controlled by Akt-mediated phosphorylation of TSC2 [31,32]. The discovery that mTORC1 is located near mitochondria and is suppressed by oxidative stress and mitochondrial dysfunction suggests that it may be involved in how damaged mitochondria induce autophagy [33,34]. The LKB1-AMPK-TSC1/2 branch seems to be important in mTORC1 suppression in response to oxidative stress in mammalian cells, despite the fact that mTORC1 may be targeted by mitochondrial signals [35].

As opposed to this, mTORC1 uses its accessory factor Atg13 to phosphorylate and inhibit ULK1, the master switch for autophagosome production [36,37]. Autophagy is triggered by simultaneous activation of ULK1 and TFEB when hunger or pharmacological inhibitors inhibit mTORC1. Under physiological circumstances, pharmacological inhibition of mTORC1 is thus considered a critical approach to activate autophagy [38]. These findings suggest that autophagy activation in response to mTORC1 inactivation is an essential cellular process regulating alkaline stress-induced cytotoxicity and alkalosis-related clinical manifestations [39]. Furthermore, EN6-mediated ATP6V1A alteration uncouples v-ATPase from Rags, resulting in suppressed mTORC1 signaling, increased lysosome acidification, increased lysosome activity, and autophagy activation. These results highlight how v-ATPase controls mTORC1 and offer a novel strategy to enhance cell clearance by covalently inhibiting mTORC1 lysosomal signaling [38].

#### 2.2.3. mTOR and Synthesis of Lipid

Adipogenesis involves the formation of adipocytes, fat-laden cells, from stem cells and their accumulation as adipose tissue in various sites in the body; both mTOR complexes affect adipogenesis. Along this line, mTORC1 may play a role in adipogenesis by regulating sterol regulatory element-binding proteins (SREBPs). In contrast, mTORC2 was previously thought to be involved in adipogenesis downstream of Akt, where early experiments in raptor knockout mice found no defects in adipogenesis [40,41]. Also, mTOR signaling is implicated in both anabolic (lipogenesis, adipogenesis, fatty acid esterification) and catabolic (lipolysis, β -oxidation) pathways of lipid metabolism. Based on these results, mTOR seems to be a good therapeutic target for treating metabolic disorders, including diabetes and obesity.

Although phosphatidic acid (PA) is generally produced via the hydrolysis of phospholipase D-catalyzed phosphatidylcholine, it may also be made by diacylglycerol kinases and lysophosphatidic acid acyltransferases, both of which are important in phospholipid biosynthesis. The sensitivity of mTOR/TOR to PA was developed to detect lipid precursors of membrane biosynthesis before doubling of cell mass and dividing [5]. In addition, PA generated through the glycerol-3-phosphate route inhibits mTORC2 activity by decreasing the interaction between rictor and mTOR, hence decreasing the effect of insulin. These results establish a connection between insulin resistance and overeating and the subsequent synthesis of triglycerides (TAGs) [42].

#### 2.2.4. mTOR and Synthesis of Proteins

Protein synthesis is a very energy-consuming process in cells. Through stimulating the synthesis of nuclear-encoded mitochondria-related proteins including TFAM, mitochondrial ribosomal proteins, and components of complexes I and V, mTOR regulates human energy consumption and mitochondrial energy production [43]. An essential amino acid diet high in leucine quickly and efficiently promotes the mTOR signaling pathway and protein synthesis in human skeletal muscle. Muscle protein synthesis and mTOR signaling are also stimulated [44].

mTOR is intimately linked to energy status and nutritional levels. In contrast, molecular chaperones and the ubiquitin–proteasome system are primarily responsible for maintaining the quality of freshly produced polypeptides. There is ample evidence indicating that the nutritional signaling system and the intracellular stress response are interrelated [45]. However, the downstream mTORC1 pathway might be activated simply by PI 3-kinase signaling. Consistent with this, GH increased Akt/PKB target site phosphorylation of TSC2, an up-stream regulator of mTORC1. To sum up, rapamycin successfully inhibited the growth hormone (GH)-induced increase in global protein synthesis in H4IIE cells [46].

#### 2.2.5. mTOR: A Master Regulator of mRNA Translation

ARNm TOP and TOP become Torin1-resistant with the loss of a single well-characterized mTORC1 substrate, the 4E-BP family of translational suppressors. By blocking the association between eIF4E and eIF4G1 binding proteins, 4E-BP inhibits translation initiation. The selective suppression of TOP and TOP-like ARNm translation by mTOR inhibition is explained by the loss of this interaction, which lowers eIF4E’s capacity to bind TOP and TOP-like ARNm much more than other ARNm [47]. Moreover, E Meyuhas and colleagues established the importance of the mTOR pathway in regulating TOP translation mitogens, GH, oxygen, and nutritional signals that all converge on the mTOR pathway to drive TOP mRNA translation [48,49,50,51].

On the one hand, 4E-BP1 inhibits eIF4E, preventing mRNA translation and proliferation and acting as a tumor suppressor. High amounts of phosphorylated and inactive 4E-BP1 have been found in different malignancies, supporting this theory. In contrast, 4E-BP1 has pro-tumorigenic properties as it promotes tumor adaptability to metabolic and genotoxic stress by selectively increasing or inhibiting the translation of particular transcripts [52]. Indeed, Thoreen et al. [47] found that mTOR inhibition causes TOP mRNAs to be preferentially displaced over eIF4F. Furthermore, overexpression of eIF4E enhances TOP mRNA translation in NIH 3T3 cells [53]. In the presence of the mTOR kinase inhibitor (Torin1) and under amino acid deprivation, Eliseeva et al. [54] showed a reduction in ACTB mRNA translation in both endogenous and reporter systems. The translation of ACTB mRNA is influenced by the degree to which the cell type is sensitive to mTOR. However, the mTOR-dependent translational response only needs the 5′ untranslated region (5′UTR) and the ACTB promoter.

#### 2.2.6. mTOR and Biogenesis Ribosome

For example, ribosome formation is one of the many anabolic processes regulated by mTOR signaling. In response to activation by mTORC1, S6K1 first becomes dissociated from the 5′ m 7 G cap on many important targets, including 40S RPS6, eIF4B, and programmed cell death protein 4 (PCD4) (PDCD4). Highly structured 5’UTR mRNAs cannot be translated efficiently without EIF4B binding to eIF4A in the pre-initiation complex and stimulating eIF4A’s mRNA helicase activity [55]. Subsequent research revealed that Ras-connected GTPases (RAGs) attract mTORC1 to the lysosome, where the small R GTPase activates it as an enriched (Rheb) homolog [56]. Recent evidence suggests that S6K1 promotes pyrimidine biosynthesis by phosphorylating a new substrate, the CAD complex, which catalyzes the first three stages of de novo pyrimidine biosynthesis through carbamoyl phosphate synthase 2, aspartate transcarbamylase, and dihydroorotase [57].

Mayer et al. [58] found that mTORC1 was also involved in rRNA precursor processing. As a result, mTORC1 orchestrates numerous steps of ribosome biosynthesis. mTORC1 regulates rRNA transcription and ribosomal protein synthesis [58]. Rapamycin inhibits the development of transcription initiation complexes involved in 45S pre-rRNA gene transcription [59]. In addition, the urb1 mutant and the mTOR and raptor morphants have decreased free ribosomal subunit synthesis and defective polysome assembly, which may be restored by overexpression of Urb1. These findings show that Urb1 regulates digestive organ development by controlling ribosome biogenesis and protein synthesis downstream of the mammalian target/mechanism of mTORC1 [60]. On the other hand, Binal et al. [61] discovered that several ribosomal proteins and translation initiation components that form the mTOR complex were overexpressed. Using the 33 genes, PPI analysis revealed a close relationship between ribosome biogenesis, translation, and mTOR signaling. Cancer stem cells (CSCs) have higher mTOR and MLST8 fluorescence levels than non-CSCs [61].

This relationship between mTOR and RanBP2 is dependent on the activity of mTOR kinase, which controls the nuclear import of ribosomal proteins. GFP-tagged ribosomal protein rpL7a has been shown to accumulate in the nucleus, suggesting that its function is dependent on the mTOR kinase. The nuclear abundance of ribosomal proteins was unaffected by rapamycin-induced mTORC1 or mTORC2 impairment, suggesting that the mTOR complex in the nuclear envelope has a special function in nuclear import [62].

## 3. mTOR and Cancer

mTOR, MAPK, and nuclear factor kappa B (NF-κB) are among the signaling pathways involved in cancer formation. The mTOR signaling system, in particular, is involved in apoptosis, cell cycle, and cancer cell proliferation [63]. Surprisingly, mTORC1 has modulated the Warburg effect, which allows cells to survive in low-oxygen environments. However, most tumors do not have TSC1 or TSC2 genetic abnormalities, and mTORC1 activation is caused by p53 inactivation mutations [64,65]. Furthermore, p53 keeps mTORC1 active through transactivation of its negative regulators, AMPK1 and TSC2 [66,67]. Downstream, the function of mTOR in carcinogenesis is coupled to various metabolic regulatory network levels activating glycolysis by boosting pyruvate kinase expression [67]. Cellular responses to existing inhibitors are diverse because of the mTOR signaling network, which includes two functionally distinct mTOR complexes, parallel regulatory pathways, and feedback loops [68].

mTOR may be phosphorylated after Akt activation, and activation of this downstream pathway may be crucial in controlling cell proliferation and differentiation, protein synthesis, and cell metabolism [69]. As already mentioned, mTOR comprises two molecular complexes; mTORC1 and mTOR2, each with its function. Indeed, mTORC2 phosphorylates additional downstream targets, which can cause Akt feedback, among other things, resulting in a limited influence on protein synthesis and cell growth. Growth hormones not only activate mTORC1, but it is also engaged in cellular stress, and it is linked to autophagy in particular [70,71,72]. Bourneville mutations cause tuberous sclerosis syndrome in the TSC protein TSC2, which suppresses mTOR action under hypoxic and energy-depleted circumstances [24,73].

Furthermore, mutations in the phosphatase and tensin homolog (PTEN) gene are the most well-known genetic abnormalities impacting mTOR signaling observed in human cancer [74]. PTEN mutations are linked to various malignancies, including endometrial cancer, melanoma, bladder, lung, breast, prostate, thyroid cancer, brain cancer, kidney cancer, and others, making it one of the most mutated tumor suppressor genes [75,76,77]. Up to 80% of human malignancies include either gain-of-function or loss-of-function mutations in either the oncogenic Raf, Ras, Akt or PI3K signaling pathways or in the tumor-suppressor genes NF1, PTEN, or TSC [78].

### 3.1. Signalling Upstream of mTOR

**Figure 2:**
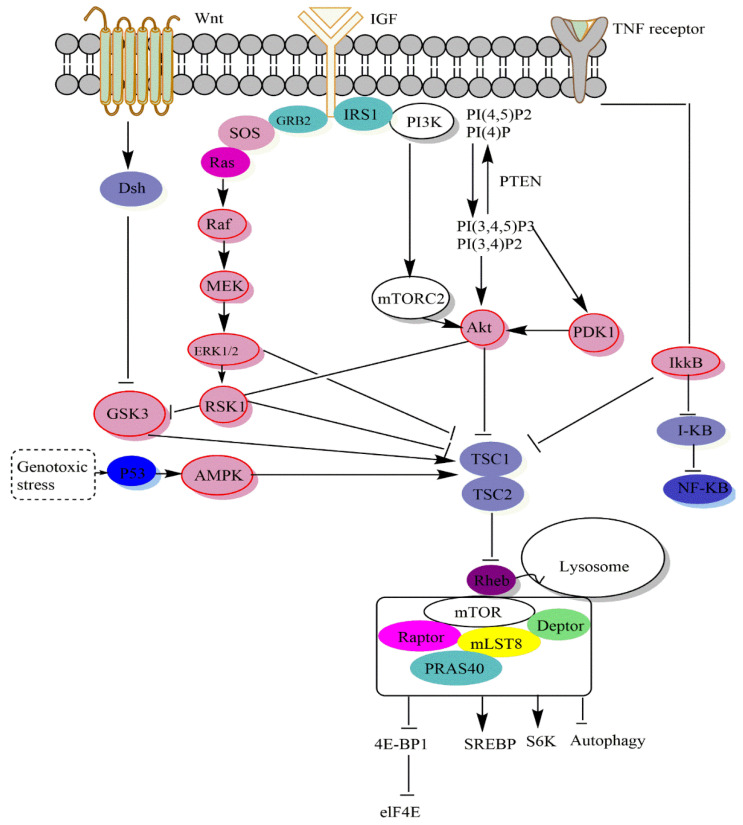
The mTOR pathway. mTORC1, also known as the rapamycin-sensitive complex, is made up of four proteins: mTOR, raptor, mLST8, and PRAS40. TSC1/2-Rheb is mTORC1’s primary upstream regulator. mTORC1 regulates protein translation by phosphorylating S6K1 and 4EBP1 via the TSC1/2-Rheb axis, which integrates cellular energy levels, growth hormones, and Wnt signals. Rheb, TSC1/2, AMPK, Rag, and Akt are the key upstream regulators of mTORC1. When mTORC1 is active at the lysosome membrane, it phosphorylates 4E-BP1, S6K, SREBP, and some autophagy components, all of which influence protein synthesis, lipid and lysosome production, energy metabolism, and autophagy. On the other hand, little is known about mTORC2’s upstream regulators.

The upstream signals they include, the substrates they control, the biological processes they oversee, and the unique protein compositions of mTOR complexes all contribute to their varying degrees of sensitivity to rapamycin [3]. Bypass methods outside the PI3K-Akt-mTOR pathway allow growth hormones to affect mTORC1 (Figure 2). By phosphorylating extracellular kinase (Erk), growth hormones like epidermal growth factor (EGF) may inhibit TSC activity and stimulate mTORC1 [79]. mTORC1 activity is regulated by many upstream signals that converge on the TSC. Akt [80], extracellular signal-regulated kinase (ERK) [79], p90 ribosomal S6 kinase 1 (RSK1) [27], IκB kinase *β* (IKK*β*) [81], and MAPKAPK2 (MK2) [82] can inhibit and phosphorylate TSC2 [28] to activate mTORC1. TSC signaling is a critical signaling pathway that controls mTORC1.

Low energy levels activate AMPK during glucose deprivation [83,84,85]. In fact, induction of tumor suppressor protein 53 (TP53), during glucose deprivation, inhibited mTORC1 via DNA damage response pathways [66]. Phosphatidylinositol-3,4-bisphosphate (PI(3,4)P2) and phosphatidylinositol-3,4,5-trisphosphate (PI(3,4,5,)P3) are two physiologically active lipid-producing proteins that are generated when phosphoinositides are phosphorylated by PI3K at the D3 location. By dephosphorylating phosphoinositides, the tumor suppressor phosphatase PTEN blocks PI3K activity at this location. As a result of its binding to the pleckstrin (PH) homology domain of the serine-threonine kinase Akt, PI(3,4,5,)P3 stimulates Akt’s membrane translocation [86,87]. TSC2 also converts Rheb to its inactive GDP-related form, which it uses to adversely regulate mTORC1 activity. Growth hormones, which activate mTORC1, reduce TSC1-TSC2 activity, while genotoxic stress, energy shortage, and oxygen starvation increase TSC1-TSC2 activity and reduce mTORC1. Rag GTPases interact with mTORC1, encouraging its translocation from the cytoplasm to the lysosomal membranes, where Rheb is thought to reside, when amino acids are present [88]. Two of the most important molecules that mTORC1 phosphorylates in order to control protein synthesis are S6K1, also known as p70 S6 kinase, and 4E-BP1, also known as eukaryotic initiation factor binding protein 4E [89].

By binding and sequencing initiation factor 4E (eIF4E), the unphosphorylated 4E-BP1 protein slows translation initiation [16,90]. In fact, by the sterol response element-binding protein (SREBP) that regulates the expression of metabolic genes responsible for cholesterol and fatty acid formation, mTORC1 stimulates de novo lipid synthesis [91]. Glycogen synthase kinase 3 (GSK3) may also activate TSC, whereas GSK3 can be inhibited by the upstream Wnt protein signal. Therefore, Wnt protein signals may activate mTORC1 in general [24,92]. Compared to mTORC1, our understanding of mTORC2 biology is still quite restricted. Growth factors activate mTORC2 by an unknown mechanism. It affects metabolism, cell survival, and cytology by phosphorylating various AGC kinases, including PKCa, SGK1, and Akt and is resistant to acute rapamycin therapy [93,94].

### 3.2. Signaling Downstream of mTOR

The overall consensus is that Akt activates mTOR, which is downstream. According to this concept, abnormal Akt activation upregulates mTOR signaling, making altered cells more responsive to rapamycin treatment [2]. Furthermore, mTOR controls protein synthesis by phosphorylating and inactivating the mRNA translation repressor, eukaryotic initiation factor binding protein 4E (4E-BP1), and phosphorylating and activating S6 kinase (S6K1) (Figure 2). These two mTOR downstream effectors, the phosphorylation of which is blocked in vivo by rapamycin, may be phosphorylated in vitro by recombinant mTOR [89,95]. Furthermore, the alanine substitution at Asp 2338 in the mTOR catalytic domain is sufficient to inhibit mTOR kinase activity against S6K1 and 4E-BP1 (in vivo and in vitro) [89,96]. In most cases, translation is controlled during the process in which a ribosome is attracted to the 5-terminus of an mRNA, which is positioned at a start codon [97].

Various translation initiation factors facilitate ribosome binding by guiding the ribosome to the 5′-end of an mRNA. The cap structure (m7GpppN, where “m” denotes a methyl group and “N” represents any nucleotide) at the 5′-end of all mRNAs generated by the nucleus is specifically recognized by the eukaryotic translation initiation factor 4E (eIF4E) [22]. Furthermore, mTOR signaling controls the phosphorylation of several proteins, the most well-known of which are those that regulate mRNA translation (Figure 2). Several locations on the eukaryotic initiation factor-binding protein 1 4E (4E-BP1) are phosphorylated. On the other hand, it is unlikely to phosphorylate Thr69/70 in 4E-BP1, even though 4E-BP1 and S6K1 connect to the raptor, mTOR’s partner [98]. Furthermore, mTORC1 regulates cellular processes, including protein synthesis, lipid synthesis, cholesterol synthesis, mitochondrial metabolism, and autophagy. Recent investigations have found that high-dose rapamycin induces apoptosis in human cancer cell lines, which is related to the suppression of 4E-BP1 phosphorylation and the dissociation of the raptor mTORC1 [99].

On the other hand, S6K activity appears to be increased in several cancer cell lines and tumors. This is often related to elevated mTOR activity caused by loss-of-function mutations in the tumor suppressors LKB1, PTEN, or TSC. It was also discovered that S6K1 levels could be increased. Amplification of chromosomal region 17q23, which encodes the S6K1 gene, is a characteristic of several malignancies, including meningioma [100,101]. Furthermore, via activating SREBP-1 and peroxisome proliferation activator (PPAR), mTORC1 stimulates lipid and cholesterol synthesis [91,102]. While mTORC1 inhibits autophagy, it is unclear why rapamycin may activate or inhibit this process, given that S6K1 has a beneficial function in autophagy induction [103]. Overexpression of S6K1 or eIF4E enhances cell size in the absence of rapamycin, and when co-expressed, they collaborate to further increase cell size. Cell growth is reduced, and the effects of eIF4E on cell size are blocked when a phosphorylation site-defective mutant of 4EBP1 is expressed, which constitutively binds to the eIF4E-Cap complex to prevent translation initiation. These findings reveal that mTOR delivers downstream signals to at least two distinct targets, S6K1 and 4EBP1/eIF4E, both of which are involved in translation regulation and govern mammalian cell growth [104].

Other studies involve mRNA in mTOR regulating expression and activities and therefore in cell transformation. Indeed, overexpression of miR-513b might dramatically reduce proliferation, invasion, migration and increase apoptosis of small-cell lung cancer (NSCLC). Overexpressing HMGB3 counteracted the effects of miR-513b on proliferation, invasion, migration, and apoptosis in non-small cell lung cancer (NSCLC). MiR-513b may control NSCLC cell proliferation, invasion, migration, and apoptosis through HMGB3, according to a study by Wang et al. [105]. In addition, Sun et al. reported that miR-99a and miR-100 suppressed mTOR in esophageal epidermal carcinoma cell lines, thereby reducing cell proliferation; thus, the miR-99a/100-mTOR signaling pathway is a potential therapeutic target to induce apoptosis to combat esophageal epidermal carcinoma cell lines [106]. Meanwhile, miR-381 overexpression suppresses RELN expression, which in turn reduces PI3K/AKT/mTOR signaling pathway activation and hence reduces prostate cancer cell growth and enhances apoptosis and autophagy in these cells [107]. Functionally, miR-216b was linked to breast cancer growth via inhibiting the mTOR signaling pathway by targeting HK2. Upregulation of miR-216b or HK2 silencing decreased cell viability, migration, and invasion while simultaneously inducing autophagy, cell cycle arrest, and apoptosis. These results suggest that miR-216b suppresses HK2 to disable the mTOR signaling pathway, thereby stopping the development of stomach cancer [108]. Both miR-100 and miR-99a target FKBP51 and IGF1R/mTOR signaling pathways in vitro, suggesting they may provide a novel therapeutic approach for the treatment of acute lymphoblastic leukemia [109].

Here, we discuss in depth the underlying mechanisms associating mTOR and different types of cancer:

### 3.3. Breast Cancer

In general, investigations have shown that mTOR signaling activation is associated with enhanced tumor progression, survival, and invasion, as well as frequently reduced survivability of the affected patient. This is particularly valid for breast cancer [110,111].

Krieger KL and colleagues conducted a study to explore how the breast cancer 1 (BRCA1)-mTORC2 interaction is functionally affected in breast cancer. The results of this investigation established that the tBRCT domain of BRCA1 interacts independently with the subunits PRR5, RICTOR, and SIN1 of the mTORC2 complex, causing disruption of the mTORC2 complex. This provides direct evidence that the DNA damage response is coordinated with pro-survival mTORC2-Akt signaling. It is also noteworthy that the RICTOR interaction affinity may be marginally greater than that of SIN1 or PRR5 [112]. Indeed, breast cancer cells not expressing functional BRCA1 have been shown to be more responsive towards mTOR antagonists. Given these insights, it appears likely that mTOR signalling is critical for BRCA1 responsiveness to DNA damage and therefore that BRCA1-negative breast cancer cells are more responsive to pan-mTOR inhibition [113].

Wazir U et al. sought to examine the relationship between the mRNA expression of mTORC1, raptor, and rictor and the development of human breast cancer. The findings showed that in breast cancer tissues (*p* = 0.0018), ductal tumors (*p* = 0.0014), in higher grade tumors (grade 2 vs. 3, *p* = 0.047) a higher expression of mTOR was identified and was related to a low overall survival (*p* = 0.01). Raptor mRNA expression was substantially increased in tumors than in normal tissues. In background breast tissue, tumors were negatively associated with Nottingham prognostic index (NPI1 vs. 2, *p* = 0.03) and tumor grade (grade 1 vs. 3, *p* = 0.01). Rictor expression was notably higher and related to better overall survival (*p* = 0.037) and disease-free survival (*p* = 0.048) [114]. Also, a strong positive relationship was observed between mTOR and hTERT (*p* < 0.00001), as confirmed in another study conducted by Dogan F et al. [115].

These findings are in accordance with the idea that mTORC1 is a major up-regulator of telomerase in breast cancer.

Although there is evidence that Akt and mTOR signaling can potentiate oncogenic signaling, research has consistently reported that the expression of Rictor, which is essential for mTORC2 signaling, is in fact decreased in breast tumors as opposed to healthy breast tissue [114].

In addition, they are two major controllers of the activity of the mTOR–raptor complex, namely the LKB1/AMPK and PI3K/Akt pathways, both of which affect the tuberous clerosis complex (TSC). Tuberin phosphorylation by Akt blocks the complex’s ability to interact with the activity of Rheb, thereby boosting the activity of mTOR [116]. These findings are supported by the fact that mutations that activate the PIK3CA gene (encoding for a PI3K subunit) occur frequently in breast cancer, typically involving mutations centered on the kinase and helical domains [117]. Other prevalent mTOR upstream mutations are found in AKT, with impairment or mutation of AKT and PTEN loss identified in breast cancer [118].

On the other hand, considering that triple-negative breast cancer (TNBC) is characterized by the lack of progesterone receptor (PR), estrogen receptor (ER), and HER2 overexpression, in this sense, Walsh et al. used an immunohistochemistry assay to measure the levels of mTOR and phospoho (p)-mTOR in 99 non-TNBCs and 89 TNBCs. They found that the nuclear protein p-mTOR was more prevalent in triple-negative cancers than in non-triple-negative cancers (*p* < 0.001), thus suggesting that mTOR could represent a novel target for TNBC management [119].

Concerning mTOR and ER, the drug tamoxifen is often indicated in patients with estrogen receptor-positive breast cancer, but they develop resistance to this drug [120]. mTOR seems to play a key function in this resistance, because ERα phosphorylates the mTOR pathway at Ser118, making it hyper-reactive to activation and therefore less susceptible to bind to tamoxifen [121]. Indeed, the PI3K/Akt/mTOR axis can be used in the long term by breast cancer cells to escape their reliance on ER signaling and consequently enhance resistance to tamoxifen [122].

Regarding mTOR and HER, and given that HER-2 expression is significant in the over-activating of mTOR signaling in breast cancer, and that HER family receptors can promote PI3K-mTOR signalling [123], resistance to HER-2 therapies in breast cancer, particularly lapatinib (a dual EGFR (HER-1 and HER-2) inhibitor) and trastuzumab (an antibody-based drug), is significantly correlated with mTOR signaling [124,125]. It should be noted that mutations in the PI3K pathway leading to activation of mTOR signaling is a factor leading to drug resistance [122].

### 3.4. Lung Cancer

Among the most widespread cancers worldwide is lung cancer. It is considered to be one of the leading causes of death, occurring in millions of individuals every year [126]. With the aim of discovering new molecular targets in the management of non-small cell lung cancer (NSCLC), research has focused mainly on the link between the PI3K/AKT/mTOR signaling pathway and lung cancer development as it affects many aspects related to cell survival, proliferation, differentiation, motility and growth [127]. The identification of this route as an important treatment target for lung cancer is primarily based on its pivotal implication in the onset and evolution of this type of cancer [128]. Deregulation of mTOR signaling is prominent in a wide range of tumors, such as lung cancer [129]. In fact, it has been reported that in NSCLC, PI3K/AKT/mTOR signaling is most commonly disrupted due to multiple molecular impairments in this pathway, such as activation of mutations and/or overactivation of receptor tyrosine kinases (RTKs), activating mutations and/or amplifications of genes involved in PI3K, and inactivating mutations or deletion of the tumor suppressor PTEN [127,128,130,131].

Furthermore, tumor suppressor liver kinase B1 (LKB1) as a negative regulator of mTOR signaling was specifically identified as being commonly mutated in lung cancer, highlighting a potential role of the mTOR pathway in the development of lung carcinogenesis [132].

On the other hand, concerning the relationship between mTOR and the expression of eukaryotic initiation factor-4E (eIF-4E) in lung cancer, there is evidence suggesting an involvement of the mTOR pathway in lung carcinogenesis via its binding to eIF-4E, acting as an oncogene in numerous studies [133]. In bronchial adenocarcinoma, eIF-4E expression was consistently reported to be increased compared to the normal lung [134]. In addition, the study by Seki et al. [135] demonstrated that the expression of eIF4E in adenocarcinomas was 3.4 to 7.4 times higher than in normal lungs and significantly correlated with tumor invasiveness and histological stage.

According to an in vivo study conducted by Frankel et al. [136], the incidence of premalignant adenomatous hyperplastic lesions was markedly enhanced due to the overexpression of IGF-1, considered an activator key to the Akt/mTOR pathway.

Similar to the Kirsten’s rat sarcoma, viral oncogene homolog (KRAS) and the epidermal growth factor receptor (EGFR) are the frequently detected gene mutation in NSCLC. In a study conducted by Conde and colleagues, they found that EGFR mutations have accumulated in a sub-group of lung cancer patients. In addition, the findings showed EGFR gene amplification in the mutant EGFR tumors, as well as a positive association between either alterations in EGFR or KRAS and mTOR signaling activation [137]. Furthermore, it was shown that in NSCLC metastases, EphA2 expression was found to be significantly higher. Indeed, the EphA2 mutation appears in lung squamous cell carcinoma and promotes invasion and survival of tumors by activation of the mTOR pathway [138].

Using immunohistochemistry and immunoblotting, the mTOR pathway was implicated in the pathobiological profiles of 150 lung carcinoma specimens and this was correlated with the downstream and upstream proteins p70S6-kinase (S6K) and Akt, respectively. In fact, mTOR and p-mTOR expressions were reported in 68.7% and 53.3% of tumors, respectively. mTor showed the strongest occurrence in lung adenocarcinomas (89.7%) [139].

### 3.5. Colon Cancer

The third most common malignancy in both men and women is colorectal cancer. It has an increased incidence of mortality with a mediocre prognosis [140]. Previous investigations have reported that hyperactivation of mTOR signaling is one of the major factors contributing to the development of colon cancer [141,142,143].

In this sense, Zhang et al. performed a study to examine the pattern of distribution of components of mTOR signaling in adenomas and colorectal cancer (CRC). The results showed that in colorectal adenomas and high-grade intraepithelial neoplasia CRC glandular elements, components of mTOR signaling, including mTOR, p70s6 K and 4EBP1, were strongly activated. Likewise, in human colorectal adenomas and cancers, ex vivo immunohistochemical studies have demonstrated that mTORC1 signaling functions as a premature signaling step in the tumorigenesis process and contributes to the evolution of healthy cells into a neoplastic state [144].

In addition to mTORC1, mTORC2 in colon cancer has also been shown to be highly overexpressed and plays an influential role in the pathogenesis of this type of cancer.

Roulin et al. found that in HT29 and LS174T colon cancer cells, rictor downregulation significantly reduces cell proliferation. as shown by cell cycle analysis, inactivation of rictor also resulted in cycle arrest at the G_1_ phase. They also noted that LS174T cells deficient in rictor were unable to generate tumors in vivo in a xenograft model [145]. Moreover, the results published by Gulhati et al. [146] confirm that high mTORC1 and mTORC2 activity contribute to regulating epithelial–mesenchymal transition (EMT), motility and metastasis of CRCs via RhoA and Rac1 signals. Targeting mTOR inhibited colon cancer growth through the 4EBP1/eIF4E/PUMA pathway [140].

Using Apc+/Delta716 Cdx2+/− compound mutant mice (a murine model of familial adenomatous polyposis), It was found in research by Aoki et al. [147] that mTOR pathway activation leads to a deregulation of translation and an enhancement of the G1-S phase, coupled with reduced p27 levels and cyclin E-Cdk2 activation. It was also observed from the results that mTORC1 promotes chromosomal instability (CIN) mediated by anaphase bridging, thereby promoting tumor initiation and progression. The anaphase bridge index (ABI) in colon cancer cells was also increased due to forced activation of mTOR via the upstream regulator Akt.

In a recent study, as shown by bioinformatic analysis, the authors have found that in colorectal tumors, the expression of E3 ubiquitin ligase protein RING finger 167 (RNF167) is decreased and that of STAMBPL1 is increased, acting synergistically to control the level of polyubiquitination of Sestrin2 in relation to leucine accessibility. Sestrin2 ubiquitination facilitates its interaction with GATOR2 and suppresses mTORC1 signaling [148].

On the other hand, there is evidence that fasting has significant antitumor benefits in different cancers, notably CRC. It has been suggested that an mTOR inhibitor may act synergistically with fasting to inhibit CRC proliferation via Farnesyl-Diphosphate Farnesyltransferase 1 (FDFT1)-mediated AKT/mTOR/HIF1α pathway inhibition [149].

Atractylenolide I (ATL-1) has been shown to be an effective drug in suppressing colorectal tumor progression, primarily by preventing colorectal cancer cell proliferation through glucose metabolism, apoptosis alteration, and stump-like behavior via the AKT/mTOR signaling pathway [150]. Furthermore, dual suppression by NVP-BEZ235 of both PI3K and mTORC1/2 signaling has been shown to lead to tumor regression in a genetically engineered mouse model for sporadic CRC [151]. In addition, there is evidence indicating that the association of PI3K/mTOR and EGFR inhibitors has the potential to enhance treatment performance in patients with KRAS-mutant CRC [152].

### 3.6. Head and Neck Cancer

Currently, the sixth most commonly diagnosed cancer worldwide is head and neck squamous cell carcinoma (HNSCC) [153]. Among many cancers, the development of head and neck cancer is the consequence of genetic changes that deregulate mTOR signaling causing a metabolic rearrangement [154]. In addition, another type of cancer, HNSCC, is characterized by hyperactivation of the mTOR pathway. Indeed, mTORC1 and mTORC2 represent contributing factors to HNSCC tumorigenesis. In HNSCC, deregulation of mTOR was identified as the most prominent genomic alteration (~80–90% of HNSCC) responsible for abnormal mitogenic signaling, compared to other established pathways such as MAPK and JAK/STAT, hosting mutations less than 10% damage [154,155].

In an HNSCCS mouse model, Sun et al. reported that conditional Tgfbr1 and Pten deletion was linked to sporadic tongue tumor formation mediated by mTOR activation [156]. Additionally, the combined targeting of mTOR and PD-L1 has been shown to enhance tumor growth suppression in a syngeneic mouse model of oral cancer [157]. Using orthotopic CAL33 xenografts carrying PIK3CA mutations, Bozec et al. investigated the effect of temsirolimus in combination with cetuximab and standard chemotherapeutic drugs such as cisplatin and 5-fluorouracil. The results showed a synergistic action of this combination leading to a nearly total arrest of tumor growth due to the profound involvement of mTOR and EGFR activity [158].

Currently, there are a few validated mutant genes responsible for the activation of mTOR signaling in HNSCC, one of the pathways affected by these mutations is the PI3K-AKT-mTOR pathway, which is activated by EGFR hyperactivation via some types of genetic and epigenetic strategies. As reported, mTORC1 plays a role in the activation of NF-κB downstream of EGFR/PI3K/Akt signaling. Mechanistically, to accelerate NF-κB signaling, mTORC1 stimulates the activity of nuclear factor kappa-B kinase (IKK) inhibitor. This was justified by the study of Li et al. in which the proliferation of head and neck squamous cell carcinoma is regulated by a positive feedback system implicating EGFR/Akt/mTORC1 and IKK/NF-κB [159].

On the other hand, the PIK3CA mutation is the frequently identified mutation in HNSCC that activates the PI3K pathway. Indeed, the study by Suda et al. [160] identified three mutations (2.6%) in PIK3CA and an amplification of the copy number was discovered in 37 cases (32.2%) for PIK3CA. The authors suggested that PIK3CA gene copy number amplification is related to a poorer prognosis in patients with HNSCC without lymph node metastasis. Keysar and colleagues analyzed HNSCC patient-derived xenografts for cancer stem cells (CSCs) and determined the molecular characteristics of tumors induced by smoking and human papillomavirus (HPV) profile (HPV-positive and HPV-negative HNSCC). This research elucidated the impact of deregulated PI3K signaling, such as enhanced SOX2 translation and ALDH expression, leading to increased spheroid and tumor formation. This investigation also noted a downregulation of SOX2 levels following AKT1 (downstream of PI3K) or EIF4E (downstream of mTORC1) silencing, indicating a potential link between SOX2 and PI3K regulation. In addition, SOX2 deletion abolished ALDH transcripts and protein level [161]. Moreover, it has been reported that the inactivation of PTEN induces hyperactivation of PI3K-mediated mTOR signaling [162]. PTEN gene mutations are also frequent in HNSCC, which may provide useful predictive markers in patients with tongue cancer [163]. Further, transforming growth factor-beta-receptor 1 (TGFBR1) may also participate in the deregulation of PI3K-mTOR signaling. Indeed, using the Cre-LoxP system, Bian et al. [164] elucidated the association of TGF-β signaling and the PI3K-mTOR pathway through conditional suppression of TGFBR1 and PTEN in HNSCC mouse models. The results showed that apoptosis decreased and cell proliferation increased, which in turn increased HNSCC tumor growth.

Moreover, mTOR signaling is reported to be aberrantly activated by HRAS mutants in HNSCC tumors [165]. In this context, the results of the study by Ruicci et al. [166] showed that mutant HRAS cells are highly resistant to PI3K suppression and suggested the involvement of MAPK and PI3K signaling pathways intersecting at ERK-TSC2, resulting in persistent mTOR action.

### 3.7. Cervical Cancer

HPV infection of the cervix has been shown to be involved in the potential for cervical cancer pathogenesis [167]. The hypothesis that mTOR gene overactivation has a significant impact on the development of human cervical carcinoma was confirmed in a study conducted by Ji et al. Using immunohistochemical analysis, the results showed significantly higher mTOR activity in cervical cancers compared to normal cervical tissue. The expression of mTOR in invasive squamous cell carcinomas of the cervix increases progressively according to the grade of malignancy. Upon activation, the downstream target p70S6K is phosphorylated by mTOR [168]. It also revealed that in malignant tissues, the expression of p70S6K was markedly increased compared to the normal cervix, and in human cervical squamous cell carcinomas it correlated to a pathological degree. Furthermore, the expression of mTOR and P70S6K was found to be strongly correlated positively [169]. The study by Faried et al. [170] aimed to investigate the prognostic and predictive function of molecular overexpression in cisplatin-based neoadjuvant chemotherapy-treated cervical cancer. The outcomes indicate that activated Akt and mTOR proved to be important predictive indicators (*p* < 0.05). The investigators also explored activated AKT (p-AKT) and mTOR (p-mTOR) expression among patients diagnosed with cervical adenocarcinoma. The findings demonstrated in 50% and 53% of cervical adenocarcinomas, respectively, that p-AKT and p-mTOR were determined. Overall, p-mTOR expression was reported to be an independent and predictive indicator of cervical adenocarcinoma.

In another study by Feng et al., morphoproteomic assays indicate constitutive activation and predominant overexpression of the mTOR pathway in both squamous intraepithelial lesions of high grade and cervical squamous cell carcinomas, as demonstrated by enhanced translocation of nuclear pmTOR and p-p70S6K, which are phosphorylated at putative activation sites, Ser2448 and Thr389, respectively. the resulting induced overexpression of the important upstream signal transducer, EGFR; and the enhanced cell cycle related correlates, Skp2 and mitotic indices [167]. In contrast, the circTPCN/miR-634/mTOR regulatory pathway has been shown to be involved in cervical cancer tumorigenesis [171]. A study carried out by Liu et al. [172] showed an ability of CD155 to interfere within a complex formed by AKT, activating the AKT/mTOR/NF-κB pathway and preventing the activation of apoptosis and autophagy in cervical cancer.

### 3.8. Reproductive Cancer

Ovarian cancer is the most prevalent malignancy of the female reproductive system. Deng et al. [173] conducted a study to determine the underlying mechanisms required in the chemoresistance of epithelial ovarian cancer (EOC). The findings demonstrated that the expression of markers of epithelial–mesenchymal transition (EMT) and cancer stem cells (CSCs) was dramatically elevated in chemoresistant EOC cells, coupled with the activation of PI3K/Akt/mTOR signaling. Similarly, overexpression of the miR-126-5p increased the growth, migration and invasion of ovarian cancer cells and suppressed their apoptosis. As evidenced by the luciferase assay, miR-126-5p was able to bind directly to PTEN. By targeting PTEN, most likely miR-126-5p could promote the PI3K/Akt/mTOR pathway [174]. Furthermore, the increase in miR-18b-5p increased the suppressive properties of exosomes on polycystic ovary syndrome progression. There is evidence that miR-18b-5p targets PTEN and may promote the PI3K/Akt/mTOR pathway [175].

Regarding prostate cancer, the results of Chang et al. reported that radioresistance in prostate cancer is related to epithelial–mesenchymal transition and improved cancer stem cell phenotypes via activation of the PI3K/Akt/mTOR signaling pathway [176]. Androgen receptor (AR), MAPK, and WNT signaling cascades are among the major interplaying oncogenic signaling cascades that may activate the PI3K-AKT-mTOR pathway and thus promote prostate cancer growth and drug resistance [177]. The deregulation of the PI3K-AKT-mTOR pathway due to the loss of PTEN is linked to androgen insensitivity and prostate cancer progression. The PI3K and AR signaling pathways are directly linked to the development of prostate cancer [178].

Furthermore, immunohistochemical analysis of prostate cancer adenocarcinoma revealed that more than 90% of the cases detected the phosphorylated form of AKT of samples and correlated with high Gleason grade of prostate cancer, confirming that aberrant activation of the PI3K/AKT/mTOR pathway is associated with the progression of prostate cancer [179].

### 3.9. Bone Cancer

Disturbances in mTOR signaling have been related to the development of malignant diseases, including bone cancer [180]. It has been published that protein expression levels of p-PI3K/p-Akt/p-mTOR were increased in the periaqueductal gray of rats with bone cancer [181]. In addition, neurofibromatosis type 1 (NF1) disease is recognized by deficiencies in bone structure combined with enhanced osteoclastogenesis. Ma et al. [182] in their investigation found that mTOR hyperactivation is involved in dysfunctional major osteoclastogenesis through hyper-proliferation using primary osteoclast-like cells (OCL) derived from a NF1 mouse model (Nf1 heterozygous mice; Nf1+/−).

On the other hand, another pathway by which activation of the mTOR pathway enhances OS cell metastasis is angiogenesis. In fact, activation of the mTOR pathway in these cells promotes cell growth and proliferation, induces cell metastasis, inhibits the intracellular processes of apoptosis, and suppresses autophagy [183]. Further research on understanding the mechanisms involved in the occurrence of bone cancer through the mTOR pathway is needed.

### 3.10. Cancers Caused by Different Type of Viruses

There is now conclusive evidence that 10–15% of all malignant tumors are caused by viruses [184,185]. In order to immortalize infected cells, HPV replicates by using the host cell’s replication machinery to express the E6/E7/E5 oncoproteins. This immortalization is carried out not only through the inhibition of p53 and Rb tumor suppressors and the reduction of apoptosis, but also, and above all, by the activation of the PI3K/Akt/mTOR pathway [186]. Researchers in the United States have demonstrated that activation of the Akt /mTOR enzyme may be identified within minutes of exposure of human keratinocytes to HPV-16 pseudovirions [170,187].

A critical metabolic sensor in the growth factor receptor (GFR) pathway, which incorporates growth factor signals into cells, is mTOR. Increased nuclear translocation of p-mTOR (Ser2448) and p70S6K (Thr389) is consistent with EGFR upstream signal transducer overexpression, enhanced cycle times and improved mitotic indices [167]. Two substrates of the mTOR complex 1, 4E-BP1 and p70S6K, are phosphorylated in response to PI3K/Akt/mTOR activation (Thr389). During the early phases of virus–host cell contact, S6K is activated, which induces the translation machinery and suppresses autophagy [188].

Shrivastava et al. [189] showed that HCV infection enhances phospho-mTOR and its downstream target 4EBP1, showing that mTOR is not a negative regulator of HCV-induced autophagy. In autophagy-deficient cells, HCV lowers phospho-mTOR, mTOR, and phospho-4EBP1. HCV upregulates Beclin1 and stimulates the mTOR signaling pathway, which may increase hepatocyte development. Conversely, Aravinth et al.[190] demonstrate that Epstein-Barr virus latent membrane protein 1 (LMP1) increases CD137 expression in Hodgkin Reed–Sternberg cell lines. CD137 expression is induced by LMP1 via the PI3K-AKT-mTOR pathway. These findings provide further evidence for the involvement of Epstein–Barr virus in the development of Hodgkin’s lymphoma.

### 3.11. Nasopharyngeal Carcinoma

Using immunohistochemistry, Wang et al. [191] investigated whether there was a correlation between the expression of the proteins p-Akt, p-p70S6K, and p-4EBP1, and the clinicopathologic features of nasopharyngeal cancer (NPC). The findings indicated that the percentage of positive protein expression for p-Akt, p-p70S6K, and p-4EBP1 in NPC was much higher than in noncancerous nasopharyngeal tissue used as control.

Increased expression of AKT, mTOR and P70S6K proteins in NPC tissues were all related to T-stage, N-stage, clinical stage, distant metastasis and differentiation compared to healthy nasopharyngeal mucosal tissues. These findings suggest a link between the AKT/mTOR signaling pathway and the development of NPCs [192].

### 3.12. DLBCL

Primary CNS lymphoma (PCNSL) is a form of aggressive non-Hodgkin’s lymphoma that occurs exclusively in the brain and spinal cord (CNS). Diffuse large B-cell lymphomas represent the vast majority of primary CNS lymphomas (DLBCLs). The positive expression levels of p-AKT, p-mTOR, p-S6, and p-4E-BP1 were substantially greater in PCNSL than in the control group. p-mTOR expression is linked to p-AKT, p-S6, and p-4E-BP1. In 18.9% of PCNSL samples, PTEN gene deletion is linked to p-AKT expression [193].

### 3.13. Different Type of Lymphomas

The capacity to target the PI3K/AKT pathway with small molecule inhibitors in Burkitt’s lymphoma cell lines was investigated by Bhatti et al. [194] who also examined the activation of this pathway in a resistant BL cell line model. PI3K/AKT activation was found to be elevated in rituximab- and chemotherapy-resistant cell lines, and inhibition of AKT or PI3K resulted in anti-lymphoma activity in vitro.

In B-cell malignancy, the B-cell receptor signaling pathway is active and the phosphoinositide 3-kinase (PI3K) pathway is the primary mediator of this activation. Additionaly, emerging PI3K blockers like idelalisib and copanlisib have demonstrated remarkable effectiveness in various types of indolent lymphomas, particularly in marginal zone lymphoma [195].

Patients with follicular lymphoma have been shown to have an unusually high concentration of somatic mutations in RRAGC, as revealed by targeted sequencing. More than 50% of mutations favored coexistence with mutations within ATP6V1B2 and ATP6AP1, both of which are part of vacuolar H-adenosine triphosphate ATPase (v-ATPase) and are reported to be needed for the activation of mTORC1 mediated by amino acids. RagC mutations improved raptor binding and made mTORC1 signaling more robust to amino acid deprivation [196].

### 3.14. Non-Mantle Hodgkin’s Cell Lymphoma (MCL)

Yu et al. [197] showed that nutlin 3A-mediated stabilization and activation of wt-p53 results in G1-S cell cycle arrest and p53-dependent death in MCL cells. They revealed that activation of p53 can significantly downregulate the AKT/mTOR route through an AMPK-dependent mechanism.

## 4. Targeting mTOR Signaling Pathways by Natural Products

Natural products have been an incomparable source of anticancer drugs (Table 1) and have potentially affected the modulation of autophagy by downregulation of the PI3K/Akt/mTOR pathway in different cancer cells [8]. This section, reported in detail the bioactive compounds that have been studied for their important activity on the PI3K-Akt-mTOR pathway in cancer. Indeed, apigetrin is a natural flavonoid glycoside found abundantly in natural products, with several activities, including anticancer property against several cancer cells, such as human gastric cancer cells, by causing cell death via several mechanisms, such as down-regulation of the PI3K/AKT/mTOR signaling pathway [7]. In another study, Xia et al. [198] reported that dihydromyricetin, a flavonoid compound extracted from *Ampelopsis grossedentata,* inhibits mTOR involved in the regulation of its upstream signaling pathways such as extracellular signal-regulated kinase, AMPK, and class III phosphatidylinositol 3-kinase/phosphoinositide-dependent protein kinase 1/protein kinase B (PI3K/PDK 1/Akt) pathways, which was induced by autophagy in HepG2 cancer cells.

Moreover, licochalcone A (LicA), another flavonoid extracted from *Glycyrrhiza inflata* Batalin root, has been shown to induce cell death and autophagy in cervical cancer cells by downregulation of the phosphatidylinositol 3-kinase (PI3K)/Akt/mammalian target of the mTOR signaling pathway [199]. In agreement with these results, piperlongumine (PL), an alkaloid isolated from *Piper longum* Linn, induced autophagy in colon cancer cells by targeting Ras proteins and PI3K/Akt/mTOR signaling cascade [200]. In the cholangiocarcinoma cell line, Atractylodin (ATD), a major compound extracted from *Atractylodes lancea*, induced autophagy by inhibiting mTOR and p38MAPK phosphorylation as well as increasing Beclin-1 expression [201]. Furthermore, cannabisin B, a bioactive compound isolated from hempseed (*Cannabis sativa* L.) hull, possesses antiproliferative activity in human HepG2 hepatocarcinoma cells by inhibiting cell growth and inducing autophagy by downregulating the activation of AKT and downstream targets of the mammalian target of rapamycin (mTOR) [202].

Similarly, induction of gastric cancer cell autophagy via downregulation of the PI3K/Akt/mTOR signaling pathway was reported with thymoquinone, as a bioactive lactone, isolated from seed oil of *Nigella sativa* [203]. Seo et al. [204] have demonstrated that curcumin, induces autophagy in human renal carcinoma Caki cells by enhancing NVP-BEZ235-induced PI3K-Akt-mTOR signaling inhibition. In addition, gallic acid has been shown to suppress Akt/mTOR signaling, leading to mitochondrial dysfunction, energy crisis, and acute myeloid leukemia cell apoptosis [205]. Gartanin, a xanthone naturally present in *Garcinia mangostana*, has shown promising anticancer properties against human urinary bladder cancer. Gartanin treatment has been reported to downregulate downstream events (p70S6 and 4E-BP1) of the mTOR pathway via two mechanisms involving AMPKα activation and AKT inactivation, respectively [206].

In addition, Kavalactones (yangonin, flavokawain A, and docetaxel), are a class of lactone compounds present in the roots of kava (*Piper methysticum* Forst), capable of inhibiting the mTOR pathway by changing the upstream (LKB1, AKT signaling, and PRAS40) and downstream (rpS6, p70S6K, and 4EBP1) events of the mTOR pathway, consistent with reduced viability of human bladder cancer cell lines [207]. Alayev et al. [208] investigated anticancer properties based on the regulation of mTOR signaling in breast cancer cell lines by exposing them to resveratrol. According to their findings, this molecule efficiently downregulated cell growth by inducing growth arrests by targeting Akt and inhibiting mTORC1 signaling pathways. This compound also activates the autophagy pathway in bladder cancer cells by modulated the Akt/mTOR signaling pathway through the regulation of microRNA expression [209].

Research findings demonstrated that glycyrrhizic acid, a bioactive compound in licorice, induces apoptosis in TF-1 leukemia cells via blocking Akt, mTOR, and STAT3 phosphorylation signaling in tumor tissues [210]. Moreover, Liu et al. [211] studied the anticancer effect of oleanolic acid, a triterpenoid that widely exists in several plant types against various cancer cell lines (A549, MCF-7, U2OS, BXPC3, PANC-1, and PC-3 cells). They revealed that this compound induces autophagy via JNK activation and mTOR inhibition that increases the resistance of cancer cells to apoptosis. Furthermore, poricoic acid A (PAA), one of the main triterpenoid compounds extracted from *Poria cocos* extract, exerted antiproliferative activity in ovarian cancer cells by downregulating the activity of the mTOR/p70S6K signaling pathway [212]. Similarly, induction of esophageal squamous cell carcinoma (ESCC) autophagy through inactivation of the AKT/mTOR signaling pathway has been reported with echinatin, a compound extracted from the Chinese herb *Glycyrrhiza uralensis* Fisch [213].

Li et al. [214] studied mTOR signaling regulation-based antiproliferative activities in human renal carcinoma cell lines (OS-RC-2 and ACHN cell) by exposing them to vitexin. They showed that vitexin treatment significantly inhibited the growth of ACHN and OS-RC-2 cells and induced apoptosis and hyperautophagy via upregulation of the AMPK/mTOR and JNK pathways and downregulating PI3K/AKT/mTOR pathways. Cryptotanshinone is a phytoconstituent isolated from *Salvia miltiorrhiza*. Bunge. This bioactive compound has been investigated for its anticancer activity against CT26 colon cancer cell lines. It has been found to induce cell autophagy and apoptosis by inhibiting the PI3K-Akt-mTOR signaling pathway, which has been proven by decreasing the expression of p-PI3K, p-Akt, and p-mTOR in colon cancer cells [215].

Yun et al. [216] demonstrated that Tanshinone IIA, as phytochemicals obtained from *S. miltiorrhizae*, mediates autophagic cell death in KBM-5 leukemia cells by the phosphorylation of AMPK and mTOR dephosphorylation as well as the activation of the Raf/ERK/p90 RSK signaling. Recently, Yang et al. [217] showed that human leukemia cancer cells were inhibited by tomentosin, a sesquiterpene lactone obtained from *Inula viscosa* (L.). Mechanistically, tomentosin induces cell autophagy and apoptosis by downregulated mTOR and p-mTOR proteins and PI3K/Akt protein expression.

In another study performed on MDA-T32 papillary thyroid carcinoma cells and a mouse tumor xenograft model, parthenolide, a bioactive sesquiterpene lactone, induced cell apoptosis via the AKT/mTOR signaling pathway, increased the expression of autophagocytic proteins, LC3-II and beclin-1, and inhibited the growth of the mouse xenograft tumors [218]. Chen et al. [219] reported that zingiberene, a monocyclic sesquiterpene, showed growth inhibition of human colon cancer cells by inducted autophagy, suppression of PI3K/ AKT/mTOR pathway, and activation of autophagy-related caspase. In pancreatic ductal adenocarcinoma, Totiger et al. [220] reported that urolithin A, a natural compound isolated from pomegranates, reduces proliferation and increases cell apoptosis. This study revealed that treatment with this compound suppresses the PI3K/AKT/mTOR pathway by reduction of the phosphorylation of AKT and p70S6K. Another compound called atractylenolide I has been reported to exert anti-tumor activity against colorectal tumor progression via inhibition of CRC cell proliferation that is mediated by the AKT/mTOR signaling pathway [150].

Arnicolide D, a sesquiterpene lactone isolated from *Centipeda minima*, has recently been shown to exert anti-tumor activity against triple-negative breast cancer cells by inhibiting the activation of Akt/mTOR and STAT3 signaling pathways [221]. Park et al. [222], has shown falcarindiol-induced apoptotic cell death via the dephosphorylation of PI3K, AKT, mTOR, and p70S6K s and autophagy via Beclin-1 expression, LC3-II conversion, and autophagosome formation in human oral squamous cell carcinoma.

Other natural products shown to trigger similar inhibitory effects on the PI3K/Akt/mTOR pathway are rotundic acid [223], eupafolin [224], chaetocochin J [225], and rhein [226].

The majority of previous studies highlighted the anticancer activity of natural products, using in vitro and in vivo models. However, clinical investigations are needed to validate the potential therapeutic efficacy of natural compounds.

**Table 1:** Effects of natural compounds in mTOR signaling pathway in cancer.

Compounds	Methods	Key Results	References
Apigetrin	AGS human gastric cancer cells	Induced extrinsic apoptosis Induced autophagy Caused G_2_/M phase cell cycle arrest through PI3K/AKT/mTOR pathway	[7]
Dihydromyricetin	HepG2 cells	Induced significantly autophagosome characteristics Promoted LC3-II and Beclin-1 expressions Suppressed mTOR activation Induced autophagy Downregulated the cell proliferation	[198]
Licochalcone A	Human cervical cancer cells	Induced mitochondria dependent apoptosis Decreased Bcl-2 expression Induced autophagy Inhibited of PI3K/Akt/ mTOR Inhibited tumor growth	[199]
Piperlongumine	DMH/DSS induced experimental colon cancer	Inhibited tumor cell growth Inhibited Ras and PI3K proteins levels Suppressed activity of Akt/NF-κB, c-Myc, and cyclin D1 Arrested cell cycle progression Induced apoptosis	[200]
Atractylodin	Cholangiocarcinoma cell line	Regulated PI3K/AKT/mTOR and p38MAPK signalling pathways Inhibited cell growth Inhibited the migration and invasion Induced autophagy Increased SB202190 and 3-MA Reduced the rate of ATD-induced autophagy Inhibited the phosphorylation of PI3K, protein kinase B/AKT, mTOR, mitogen-activated protein kinase (p38MAPK) Elevated Beclin-1 expression and LC3 conversion Decreased p-AKT/AKT, p-mTOR/mTOR, and p-p38MAPK/p38MAPK	[201]
Cannabisin B	HepG2 human hepatoblastoma cells	Induced cell death Induced S phase cell cycle arrest Inhibited survival signaling Blocked AKT activation Down-stream mTOR targets	[202]
Thymoquinone (2-isopropyl-5-methyl-1,4-benzoquinone)	Gastric cancer cells (MGC80-3 and SGC-7901)	Exhibited significant growth inhibitory effects Inhibited cell migration ability Downregulated mesenchymal gene expression Inhibition of PI3K/Akt/mTOR signalling pathway key proteins	[203]
Curcumin	Human renal carcinoma caki cells	Downregulated Mcl-1 protein expression Decreased Bcl-2 mRNA and protein expression Induced apoptosis	[204]
Gallic acid	Acute myeloid leukemia (AML)	Induced apoptosis Inhibited mitochondrial respiration Reduced ATP production and oxidative stress	[205]
Gartanin	Human urinary bladder cancer cell lines (T24 and RT4)	Suppressed p70S6 and 4E-BP1 expressions Inducted autophagy Downregulated Bcl-2 expression Activated the p53 pathway	[206]
Kavalactones yangonin	Bladder cancer cells (RT4, T24, UMUC3, HT1376, and HT 1197 cell lines)	Elevated beclin and ATG5 expression Increased LKB1 expression Decreased the phosphorylation of Akt, PRAS40, rpS6, p70S6K, and 4E-BP1 Reduced the viability of bladder cancer cell lines	[207]
Resveratrol	Breast cancer cell line	Downregulated cell growth Prevented mTORC1 signaling pathways Activated the autophagy pathway Modulated Akt/mTOR signaling pathway Regulated microRNA expression	[208]
Resveratrol	Bladder cancer cell line	Activated the autophagy pathway Regulated microRNA expression Modulated the Akt/mTOR signaling pathway	[209]
Glycyrrhizic acid	TF-1 leukemia cells	Induced apoptosis Blocked Akt, mTOR, and STAT3 phosphorylation signaling	[210]
Oleanolic acid	Cancer cell lines (A549, MCF-7, U2OS, BXPC3, PANC-1, and PC-3 cells)	Induced autophagy Activated JNK Inhibited mTOR	[211]
Poricoic acid A	SKOV3 ovarian cancer cells lines	Suppressed SKOV3 cellular viability, migration, and invasion Induced SKOV3 cell apoptosis Increased LC3-II/LC3-I ratio Inhibited mTOR and p70s6k phosphorylation Reduced the xenograft tumor weight	[212]
Echinatin	Esophageal squamous cell carcinoma (ESCC)	Inducted autophagy Inactivated the AKT/mTOR	[213]
Vitexin	Human renal carcinoma cell lines (OS-RC-2 and ACHN cell)	Inhibited cell growth Induced apoptosis and hyperautophagy Upregulated the AMPK/mTOR and JNK pathways Downregulated PI3K/AKT/mTOR pathways	[214]
Cryptotanshinone	CT26 colon cancer cell lines	Induced cell autophagy and apoptosis Inhibited PI3K-Akt-mTOR signaling pathway Diminished p-PI3K, p-Akt, and p-mTOR expressions	[215]
Tanshinone IIA	KBM-5 leukemia cells	Induced autophagic cell death Induced AMPK phosphorylation Induced mTOR dephosphorylation Activated the Raf/ERK/p90 RSK signaling	[216]
Tomentosin	Human leukemia cancer cells	Induced cell autophagy and apoptosis Downregulated mTOR and p-mTOR proteins and PI3K/Akt protein expressions	[217]
Parthenolide	MDA-T32 papillary thyroid carcinoma cells and mouse tumor xenografts	Induced cell apoptosis Increased LC3-II, and beclin-1 expression Inhibited the mTOR/PI3K/AKT cascade Inhibited mouse xenograft tumor growth	[218]
Zingiberene	Human colon cancer HT-29 cell line	Inhibited colon cancer cell proliferation Induced autophagy Increased LC3-II expression Decreased p62 expression Inhibited mTOR/PI3K/AKT signalling pathway	[219]
Urolithin A	Pancreatic ductal adenocarcinoma (PDAC)	Inhibited PDAC cell proliferation and migration Enhanced apoptosis Downregulated PI3K/mTOR pathway Suppressed pancreatic tumor growth Reduced phosphorylation of AKT and p70S6K	[220]
Rotundic acid	Human hepatocellular carcinoma cell lines (HepG2, SMMC-7721) HepG2 xenograft mouse model	Inhibited HCC cell proliferation Induced cell cycle arrest, DNA damage, and apoptosis Inhibited tumor growth Regulated the expression of the proteins involved in PI3K/AKT/mTOR and MAPK pathways	[223]
Arnicolide D	MDA-MB-231 and MDA-MB-468 triple-negative breast cancer (TNBC) cell lines	Reduced cell viability Induced G_2_/M cell cycle arrest and apoptosis Reduced cell viability Induced G_2_/M cell cycle arrest and apoptosis	[221]
Atractylenolide I (ATL-1)	COLO205 and HCT116 CRC colorectal cancer cell lines	Inhibited CRC cell invasion Downregulated the phosphorylation of proteins related to the AKT/mTOR pathway	[150]
Falcarindiol	Human oral squamous cell carcinomas (OSCCs) cell lines	Suppressed cell growth Induced apoptosis Induced PI3K, AKT, mTOR, and p70S6K dephosphorylation Induced autophagy Induced antimetastatic effects	[222]
Eupafolin	Breast cancer cell lines	Decreased p-PI3K, p-Akt, and p-mTOR protein levels Increased apoptosis rate Increased the protein levels of Bax and cleaved caspase 3 Decreased Bcl-2 Inhibited cell migration and invasion Promoted cell apoptosis Induced G_0_/G_1_ phase arrest	[224]
Chaetocochin J	Colorectal cancer cell lines SW480, HCT116, and RKO	Induced apoptosis and growth inhibition Induced apoptosis and autophagy Inhibited PI3K/AKT/mTOR signaling pathways	[225]
Rhein	Human CRC cell lines HCT116, HCT15, and DLD1 and xenograft mice model	Inhibited cell growth Induced S phase cell cycle arrest and apoptosis Inhibited CRC cell migration and invasion ability Suppressed the mTOR/p70S6K signaling pathway	[226]

## 5. Synthetic Compounds as Cancer Preventive and Therapeutic Agents by Targeting the mTOR Signaling Pathway

As described above, activation of the mTOR pathway may play a role in tumorigenesis. Following knowledge advances in the mechanism of mTOR activation and in the context of developing new agents, several in vitro and in vivo research works (Table 2) have shown that the tumoral proliferation could be inhibited by the inhibitors of mTOR (Table 2). Indeed, Karbowniczek et al. [227] reported that rapamycin inhibits cell growth and expansion. In preclinical studies, everolimus and temsirolimus, two rapamycin analogs, induced decreased angiogenic capillary perfusion and cytostatic tumor growth inhibition.

In clinical trials, everolimus, approved by the FDA, did not exert this efficacy in treating patients with metastatic melanoma [228]. In a new phase II study of everolimus in patients with advanced melanoma characterized by mTOR mutations [229]; the results revealed that patients with mTOR-mutated melanoma respond significantly better, whereas mTOR inhibitors have a limited effect in patients with unselected melanoma. The authors of this study suggest that it may be possible for future prospective studies to determine appropriate subjects who would respond to treatment with mTOR inhibitors. In addition, another research work reported, in a phase 2 trial from the Sarah Cannon Oncology Research Consortium, by Hainsworth et al. [230], that the everolimus/bevacizumab combination was tolerable and exerted a moderate effect on patients with metastatic melanoma. However, the temsirolimus/temozolomide combination increased apoptotic death, leading to a significant decrease in tumor growth and melanoma cells. Additionally, in a phase I clinical study, the combination of hydroxychloroquine with temsirolimus accelerated cell death in melanoma [231,232]. In animal models, another compound namely VS-5584, with a high-affinity dual PI3K and mTOR inhibitor, is well tolerated [233,234]. Indeed, in vitro and in vivo investigations of the effect of this compound against melanoma cells have demonstrated its important potency as an excellent PI3K/mTOR dual inhibitor [235,236,237].

Interestingly, oral administration in nude mice of this low-molecular-weight compound suppresses the growth of A375 melanoma xenograft. Moreover, the co-administration of VS-5584 with ABT-737 reinforces this effect; therefore, clinical evaluation of VS-5584 in melanoma patients is recommended [236]. In another study carried out in melanoma models, SKLB-M8, a millepachine derivative, showed inhibition of proliferation and induced apoptosis via AKT/mTOR signaling pathway and inhibited angiogenesis with a decrease in ERK1/2 phosphorylation [238]. Itraconazole is an antifungal used to treat fungal infections. This azole compound is FDA-approved to treat some cancers, including melanoma [239,240]. According to Liang et al. [240], this molecule is a potent inhibitor of the Hedgehog/Wnt and PI3K/mTOR pathways.

In 2021, the FDA approved albumin-bound rapamycin under the brand name Fyarro™ as an injectable suspension to treat perivascular epithelioid cell tumor which has grown or spread and cannot be treated by surgery [241]. In 2022, FDA registered the recent mTOR inhibitor for clinical use under the name Hyftor™ of which rapamycin is an active compound in this substance to treat facial angiofibroma linked with tuberous sclerosis in children over 6 years of age and adults [241].

**Table 2:** List of synthetic compounds targeting mTOR signaling pathway which have been approved by the United States Food and Drug Administration (FDA) [241].

Inhibitors	Brand Name	Drug’s Form	Administration	Year of FDA Approved	Conditions
Rapamycin	Rapamune	Tablets or solution	Oral route	1999	Lymphangioleiomyomatosis, a rare lung pathology known by abnormal proliferation of smooth muscle-like cells and lymphatic treatment of facial angiofibroma linked with tuberous sclerosis.
	Hyftor™	Gel	Topical route	2022	
Everolimus	Afinitor^®^	Tablets	Oral route	2009	Advanced hormone receptor, epidermal growth factor receptor breast cancer; progressive neuroendocrine tumors of pancreatic origin; progressive neuroendocrine tumors of lung origin or gastrointestinal; subependymal giant cell astrocytoma and renal angiomyolipomas linked with tuberous sclerosis; advanced renal cell carcinoma.
	Disperz^®^	Tablets for suspension	Oral route	2012	
	Zortress^®^	Tablets	Oral route	2010	
Temsirolimus	Torisel^®^	Solution	Intravenous route	2007	Advanced renal cell carcinoma
Nanoparticle rapamycin bound to albumin	Fyarro™	Suspension	Intravenous route	2021	Treatment of perivascular epithelioid cell tumor

## 6. Immunotherapy Targeted mTOR

### 6.1. mTOR and Immunological Cells

Because it adapts to many external variables, such as growth hormones, energy, amino acids, and oxygen levels, the mTOR signaling governs cell growth and proliferation, survival, protein synthesis, and glucose intake [37]. The mTOR, in regulating many facets of cancer, including cell cycle, survival, metabolism, motility, and genomic instability, has been found in numerous studies, and mTOR signaling is generally aberrantly active in many malignancies [37,155]. Consequently, therapeutic reagents to block this pathway in oncology have been developed, and scientists have started to recognize it as a crucial target for cancer therapy [242,243]. Recent studies have also proven that mTOR plays an important role in immune system modulation (innate and adaptive immunity). For example, T cells, TAMs, and antigen-presenting cells are all regulated by mTOR, which promotes their development and activation [9]. In addition, studies have demonstrated the modulating effects of mTORC1/2 on diverse human cells such as CD4, CD8, Treg, tumor-associated macrophages TAM, cancer-associated fibroblasts CAF, endothelial cells, and MDSCs (myeloid-derived suppressor cells) [10,11].

mTORC1 is a critical link between metabolism and immune function as it regulates the metabolic programs that lymphocytes require for fast proliferation and controls the immunological signals [244]. Investigations have revealed that the role of mTOR in adaptive immunity is multifaceted and can stimulate or inhibit immune responses. In this context, new evidence suggests that the anticancer effects of mTOR inhibitors are partly mediated by the adaptive immune response [245].

### 6.2. T Cells

Cytotoxic cells are well-known in the human system for their ability to target cancer cells and kill them while also maintaining a memory response [244]. Recently, mTOR inhibition has been shown to modulate viral infection-induced CD8+ T cells by regulating memory CD8+ T-cell formation [246]. Furthermore, Polizzi et al. [246] found that mTORC1 and mTORC2 may play distinct roles in CD8+ cell regulation. mTORC1 favorably influences CD8+ T-cell effector responses and glycolytic phenotype while mTORC2 activity is engaged in the upregulation of CD8+ T-cell memory. mTOR increases the metabolic activity of T cells [247]. mTORC1 and mTORC2 assist CD4+ T-cell commitment and activation [248]. In vitro or in vivo, an upstream activator of mTORC1 did not develop into Th1, Th17, or Th2 effector cells [248,249]. Furthermore, mTOR drives the glycolytic program in antigen-stimulated T cells by promoting the production of MYC and HIF-1, which in turn mediate the activation of glycolytic enzymes and transporters [250,251]. In metastatic prostate cancer patients, Templeton et al. [252] found that treatment with everolimus (mTOR inhibitor) leads to a decrease in CD4+ cells and an increase in Treg cells, as well as an increase in progression-free survival.

Tregs have long been known to suppress anti-tumor immune responses through various mechanisms, including IL-10 production and IL-2 sequestration [247,253]. Treg growth is induced by reduced mTOR signaling via FOXO3 expression; Tregs with the forkhead box 3+ (FOXO3+) gene play a crucial role in tumor immunity by suppressing effector T cells [254]. Unlike CD4+ T cells, Tregs have higher FOXO3 expression [255]. Low CD8+ cell count in tumor infiltrate and increased Treg cell count in tumor infiltrate are linked to poor prognosis [245]. Furthermore, stimulation of mTORC2 in PTEN-deficient Tregs leads to a decrease in their stability and differentiation ability [256,257]. mTORC1 has also been shown to be required to program Treg-suppressive properties [258].

### 6.3. TAMs

TAMs are a type of immune cells present in large numbers in tumors and are attracted by soluble factors (cytokines and chemokines) or produced from tissue-resident macrophages [259]. mTOR has also been shown to play a role in macrophage activation and differentiation [260]. mTORC1 downregulation by various pharmacological and genetic techniques leads to both decreased pro-inflammatory cytokine production by macrophages, with a corresponding reduction in inflammation, and imbalances in macrophages’ M1 polarization, according to many studies [261,262]. TSC1 deletion in macrophages causes constitutive mTORC1 activation and decreased IL-4 production, inducing M2 polarization [263].

### 6.4. MDSCs

MDSCs are a diverse population of immature myeloid cells that include macrophage, granulocyte, and dendritic cell progenitors at different developmental stages (DC) [264]. Both cancer cells and MDSCs use mTOR signaling to modulate MDSC recruitment in TME. Indeed, the mTOR axis promotes the generation of soluble components important in MDSC recruitment in cancer cells, whereas mTOR signaling modulates the expression of a particular antigen on the surface of MDSCs [265,266]. Welte et al. [267] found that mTOR signaling enhances MDSC formation in breast cancer cells by upregulating granulocyte colony-stimulating factor (G-CSF). Indeed, rapamycin treatment and raptor suppression reduce G-CSF levels [267]. In addition to (G-CSF), TGF- plays a role in the recruitment of MDSCs in TME and the expression of CD39/CD73 [268], with mTOR playing a vital role in the regulation of this expression. Several investigations on mice have shown that the production of these two ectonucleosidases causes tumor cells to evade cytotoxic T-cell responses [265].

### 6.5. Combination of mTOR Inhibitors with Therapies

Cancer immunotherapy has shown great clinical success in treating many types of advanced cancers in the recent period [269]. Notably, treatments with monoclonal antibodies PD-1 and PD-L1 showed the greatest clinical benefit [270,271,272]. On the other hand, other studies have shown that many patients who respond to PD-1, PD-L1 immunotherapy develop acquired resistance to immunotherapies over time [273], prompting researchers to consider a variety of other strategies and combination therapies to improve the efficacy of cancer immunotherapies in larger numbers of patients [274,275], as well as to investigate standard variables that influence immunotherapy efficacy, such as T-cell production, effector function mediation, and the establishment of stable immunological memory [276,277,278]. Based on its influence on human cells, PI3K-AKT-mTOR inhibition and its combination with various therapeutic techniques have shown high efficacy in cancer therapy [277,279,280].

Table 3 summarises mTOR inhibitors in combination with other therapies in different types of cancer (Table 3). As can be seen, different studies investigated the combinatory effects between mTOR inhibitors and other therapies.

### 6.6. Inhibition of mTOR in Combination with Tumor Vaccines

Various cancer vaccines have been developed to improve tumor antigen-specific T lymphocyte activation [292]. Although the scope of these techniques does not always correlate with tumor regression, they, however, contribute to the generation of systemic, tumor-specific T-cell responses [282,293]. For this reason, many preclinical investigations on the effects of combining mTOR inhibitors with cancer vaccines have been conducted [282,294,295]. Within this context, Wang et al. [281] investigated the effect of temsirolimus on heat-shock protein-based vaccinations in mice with pre-existing renal cell cancer and melanoma. These researchers found that administering the vaccine and temsirolimus to mice for 60 days reduced tumor growth [281]. While vaccine treatment alone significantly reduces tumor development, this suggests that temsirolimus improves cancer vaccine efficacy [281]. Similarly, a recent study found that tumor-specific CD8 T cells produced by the combined therapy (vaccine + temsirolimus) have higher expression of CD127 and CD67L (phenotypic features of central memory CD8 T cells) than those induced by vaccination alone [291].

Diken et al. [282] showed that rapamycin improves the therapeutic effect of antigen-specific CD8+ T-lymphocytes generated upon vaccination with antigen-encoding RNA. In addition, the survival of mice given a combination treatment of RNA vaccine and rapamycin was much longer (91.5 days) than those given only one of these drugs (32 and 46 days, respectively) [282]. Moreover, Yu-li Chen et al. [293] investigated the possibility of suppressing mTOR to improve the anti-tumor effects of DNA vaccines by modulating DC activities. Mice survival was dramatically increased in the groups that received the vaccine and rapalog when injected with TC-1 tumor cells. In tumor-draining lymph nodes, treatment with vaccination and everolimus or rapamycin dramatically stimulated E7-specific interferon (IFN)-secreting CD8+ T cells. Similarly, mice injected with OVA-expressing B16 melanoma cells and immunized with dendritic cells previously pulsed with lipopolysaccharides (LPS). OVA and rapamycin showed a much slower tumor growth compared to dendritic cells treated with LPS and OVA or LPS alone [293]. Following immunization with rapamycin-treated dendritic cells, OVA-specific CD8+ tumor-infiltrating lymphocytes were stimulated in harvested tumors [296]. These results suggest that the administration of mTOR inhibitors in combination with other anti-tumor vaccines has a positive impact.

However, it has always been noted that the dose and schedule of mTOR inhibitor administration are critical to tip the balance towards immunostimulatory effects [297,298]. Nonetheless, one study found that adding rapamycin to a cancer vaccination had negative implications at high or low doses, highlighting the need for further research and identification of optimal therapeutic regimens that are likely not limited to quantity and time [283,299]. Targeting mTOR combination with cytotoxic T-lymphocytes-associated protein 4 CTLA-4 inhibits T cell proliferation by competing with the co-stimulatory molecule CD28 for binding to CD80 and CD86 ligands [300]. The combination of rapamycin and CTLA-4 inhibition demonstrated promising results. Anti-CTLA-4 and rapamycin treatment during T-cell priming resulted in a considerable decrease in tumor growth and a significant increase in tumor-free survival in mice compared to either therapy alone [301]. The frequency of antigen-specific CD8+ T cells in peripheral blood leucocytes increased significantly due to this action [301].

### 6.7. mTOR Inhibitors and Immune Checkpoint Modulation

The regulation of programmed cell death receptor 1 (PD-L1) has been the subject of several investigations in treating various cancers. PD-1 is extensively expressed in Treg cells, whereas PD-L1 is widely expressed in non-hematopoietic stromal cells and malignancies [302,303]. PI3K/AKT/mTOR is one of the pathways that regulate PD-L1 as in NSCLC [286]. In this respect, Lastwika et al. [304] found that activating the mTOR pathway regulates PD-L1 expression in vitro and in vivo in lung carcinoma. Moreover, work by Mittendorf et al. [305] indicated that treatments with rapamycin, an mTOR inhibitor, dramatically reduce PD-L1 mRNA transcripts in PTEN-mutant triple-negative breast cancer cell lines.

Numerous studies have been conducted to evaluate the effect of combining mTOR inhibitors with other immunotherapies in different types of cancer. In this line, Moore et al. [157] examined the effects of combining mTOR inhibitors with anti-PD-L1 monoclonal antibodies (mAb) in mice with MOC1 tumors and compared the results with mice treated with anti-PD-L1 mAb alone. These researchers found that after ex vivo treatment, the first group had better long-term primary tumor control and survival and an increased expansion of peripheral antigen-specific CD8 T cells, tumor-infiltrating NK cells, and IFN production. Moreover, research findings showed that rapamycin and anti-PDL1 mAb are reduced when CD8+ T cells are depleted but not NK cells, indicating the importance of CD8+ T cells in this pathway [157,296]. In hepatocellular carcinoma, researchers have described the effect of combining mTOR inhibitors with anti-PD-1 antibodies [287]. In immunosuppressed NOD/SCID mice, a combination of mTOR kinase inhibitor INK128 with PD-1 inhibition dramatically reduced hepatocellular carcinoma growth compared to solo therapy [287]. Similarly, a combination of mTOR kinase inhibitors with immune-checkpoint blockers such as anti-PD-1, anti-PD-L1, or anti-CTLA-4 significantly reduced MC38 or CT-26 tumor development [266]. Moreover, in CT26 tumors, this combination reduced the fatigue profile of tumor-infiltrating lymphocytes (TILs) while increasing IFN-expressing CD8+ T cells [266]. Another study investigated the effects of an mTOR kinase inhibitor in combination with a CD40 agonist antibody in a metastatic renal cell carcinoma [288].

Combination therapies targeting the PD-1 and CTLA-4 immune checkpoints have shown the greatest therapeutic efficacy in improving the effector function of tumor-specific T cells [12,13]. Anti-CTLA4 therapy promotes tumor-specific CD8+ T cell proliferation in secondary lymphoid organs. In contrast, anti-PD1 treatment reduces tumor-specific CD8+ T cell-fatigue in tumor tissue, hence increasing the quantity and function of tumor-specific effectors [12]. Unfortunately, since its introduction into clinical practice, it has become clear that these combined therapies are handicapped due to primary or acquired resistances [306]. Generally, failure of anti-cancer therapy is caused by different factors, including defects in antigen presentation, resistance of T cells, secretion of immunosuppressive cytokines, and the presence and activity of MDSCs and Tregs in tumor tissue [307,308]. Therefore, inhibition of PI3K-AKT-mTOR might interact to improve the effectiveness of this combination by increasing the secretion of immunosuppressive cytokines [289,295], MDSC, and Treg infiltration into tumor tissue, and enhancing memory T cell formation [220,265,295]. Thus, combining therapeutic techniques involving PI3K-AKT-mTOR inhibition with anti-PD-1/anti-CTLA4 immunotherapies is highly beneficial.

## 7. Conclusions and Perspectives

This work highlighted the upstream and downstream mTOR signaling pathways and their significant implications in tumor transformation. Indeed, mTOR plays an important role in several physiological processes, including cell proliferation. The activation of mTOR is correlated with the development of tumors, particularly in leukaemias. Through its implications in the cancerization program, mTOR is suggested to be a valid cancer biomarker. Furthermore, the inhibition or blockade of mTOR and its signaling pathways is considered a primary therapeutic strategy for certain cancers. In addition, we have demonstrated in this work that drugs of both natural and synthetic origin have shown the ability to inhibit mTOR. Furthermore, mTOR-based immunotherapy was studied by several researchers and showed remarkable results. Molecules and immunological strategies targeting mTOR can be used as antitumor drugs against mTOR-related human cancers. However, further studies need to be conducted to clarify the pharmacodynamic actions of each active compound. Moreover, pharmacokinetic selectivity should also be investigated in animal and human experiments. Future work should focus on finding novel pharmacological approaches to this pathway that promise to extend lifespans, as this pathway is not yet fully understood.

## Figures and Tables

**Table 3:** mTOR inhibitors in combination with other therapies in different types of cancer.

Study	Cancer	Conclusion	References
Temsirolimus, (mTOR inhibitor) in combination with heat shock protein cancer vaccines	Renal cell carcinoma (RCC) and melanoma (B16)	Temsirolimus enhanced the anti-tumor activity of cancer vaccines. The enhanced anti-tumor activity associated with temsirolimus was immune-mediated.	[281]
The impact of rapamycin on the in vivo primed RNA vaccine	B16 melanoma	Treatment with rapamycin skews the vaccine-induced immune response toward the formation of a superior memory pool and results in a better response.	[282]
Impact of rapamycin on the antitumor efficacy of a human papilloma virus E7 peptide vaccine (CyaA-E7)	Cervical cancer TC-1	In animals vaccinated with CyaA-E7, rapamycin administration had a negative impact on the recruitment of CD8+ T cells into TC-1 tumors along as well as the ability of the vaccine to reduce infiltration of T regulatory cells.	[283]
Effect of mTORC1/2 dual kinase inhibitor vistusertib (AZD2014) in combination with anti-CTLA-4 (αCTLA-4), αPD-1 or αPD-L1 immune checkpoint blockade	MC-38 or CT-26	Vistusertib/immune checkpoint combination has been shown to reduce the occurrence of exhausted phenotype tumor-infiltrating lymphocytes (TILs), as well as increasing frequencies of activated Th1 polarized T-cells in tumors.	[284]
Study of Treg control in addition to mTOR inhibition in preclinical models.	B16-gp100	The combination therapy enhanced CD8 memory formation and function.	[285]
Control of PD-L1 Expression by oncogenic activation of the AKT-mTOR pathway in non-small cell lung cancer	Lung adenocarcinomas and squamous cell carcinomas	In vitro and in vivo PD-L1 expression is regulated by the activation of the AKT-mTOR pathway. It has been demonstrated that mTOR is required for PD-L1 induction by both oncogenic and IFN-mediated pathways.	[286]
Combining mTOR and PD-L1 inhibition improves tumor control in syngeneic oral cavity cancers	Oral cancer	Rapamycin has been found to increase IFNγ production capacity in peripheral and tumor-infiltrating CD8 T cells.	[157]
The combination of rapamycin and programmed cell death-1 (PD-1) checkpoint inhibition prevents the growth of hepatocellular carcinoma	Hepatocellular carcinoma	Targeting mammalian target of rapamycin pathways in combination with PD-1 may result in increased antitumor efficacy in cancer patients.	[287]
Combination of αCD40 agonistic antibody and the ATP-competitive mTOR kinase inhibitory drug AZD8055 elicited synergistic anti-tumor responses.	Metastatic renal cell carcinoma	When combined with CD40 therapy, the ATP-competitive mTOR kinase inhibitor AZD8055 can cause a remodeling of the tumor immune milieu and result in the regression of a metastatic cancer that has already spread.	[288]
Lewis lung cancer mouse model: synergistic effects of PD-1 inhibition and endostar on PI3K/AKT/mTOR-mediated autophagy and angiogenesis	Lewis lung carcinoma	IFN-secretion increased, myeloid-derived suppressor cell (MDSC) accumulation decreased, pro-inflammatory cytokine IL-17 and immunosuppressive factor TGF-1 levels fell, and CD8+ T cell suppression was reversed as a result of this synergistic impact.	[289]
PI3K/AKT/mTOR pathway targeting natural compound urolithin A for pancreatic cancer.	Pancreatic ductal adenocarcinoma (PDAC)	Treatment of PDAC cells with Uro A improved survival of Ptf1aCre/+; LSL-KrasG12D/+; Tgfbr2flox/flox (PKT) mice compared to vehicle or gemcitabine therapy alone, successfully suppressed the formation of tumor xenografts, and prevented the phosphorylation of AKT and p70S6K in vitro.	[220]
Comparison of the powerful second-generation mTOR inhibitor MLN0128 with the drug temsirolimus	RCC growth and metastasis	Our findings showed that MLN0128 outperformed temsirolimus in preventing both metastases and primary RCC growth, providing strong evidence in favor of additional clinical research into dual mTOR inhibitors for the treatment of RCC.	[290]
Examined the interaction between temsirolimus and anticancer vaccinations. using a range of cancer vaccine designs (short and long peptides or the B subunit of Shiga toxin as an antigen delivery vector)	Melanoma, lung, and colon cancer.	We demonstrated that temsirolimus treatment effectively reduced tumor development and improved tumor-specific CD8 T-cell responses brought on by vaccination.	[291]
